# Extended Sampling of Macromolecular Conformations from Uniformly Distributed Points on Multidimensional Normal Mode Hyperspheres

**DOI:** 10.1021/acs.jctc.4c01054

**Published:** 2024-12-12

**Authors:** Antoniel A. S. Gomes, Mauricio G. S. Costa, Maxime Louet, Nicolas Floquet, Paulo M. Bisch, David Perahia

**Affiliations:** † Laboratório de Física Biológica, Instituto de Biofísica Carlos Chagas Filho, Universidade Federal do Rio de Janeiro, Rio de Janeiro 21941-902, Brazil; ‡ Laboratoire de Biologie et Pharmacologie Appliquée (LBPA), UMR 8113, CNRS, École Normale Supérieure Paris-Saclay, Gif-sur-Yvette 91190, France; § Institut des Biomolecules Max Mousseron, UMR 5247, CNRS, Université de Montpellier, ENSCM, Montpellier Cedex 05 34095, France; ∥ Programa de Computação Científica, Vice-Presidência de Educação Informação e Comunicação, Fundação Oswaldo Cruz, Rio de Janeiro 21040-900, Brazil

## Abstract

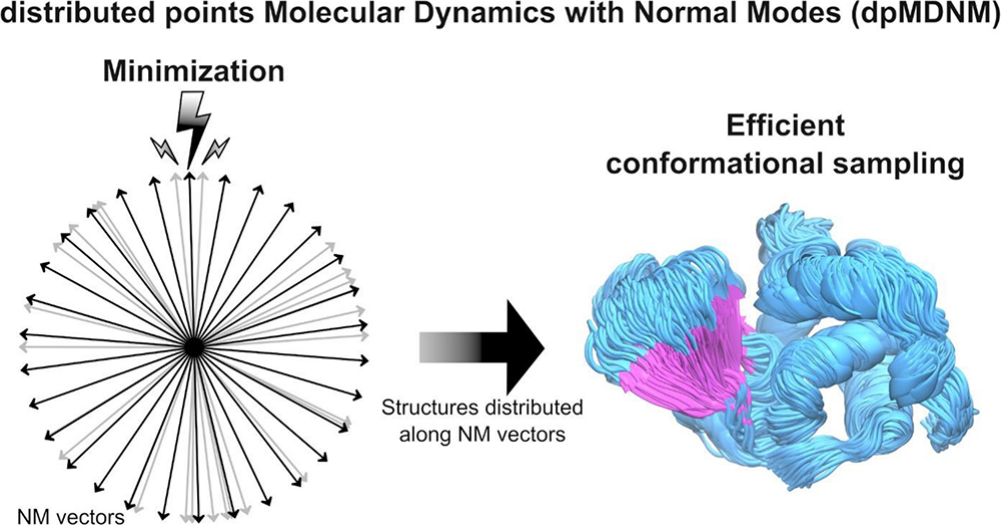

Proteins are dynamic entities that adopt diverse conformations, which play a pivotal role in their function. Understanding these conformations is essential, and protein collective motions, particularly those captured by normal mode (NM) and their linear combinations, provide a robust means for conformational sampling. This work introduces a novel approach to obtaining a uniformly oriented set of a given number of lowest frequency NM combined vectors and generating harmonically equidistant restrained structures along them. They are all thus uniformly located on concentric hyperspheres, systematically covering the defined NM space fully. The generated structures are further relaxed with standard molecular dynamics (MD) simulations to explore the conformational space. The efficiency of the approach we termed “distributed points Molecular Dynamics using Normal Modes” (dpMDNM) was assessed by applying it to hen egg-white lysozyme and human cytochrome P450 3A4 (CYP3A4). To this purpose, we compared our new approach with other methods and analyzed the sampling of existing experimental structures. Our results demonstrate the efficacy of dpMDNM in extensive conformational sampling, particularly as more NMs are considered. Ensembles generated by dpMDNM exhibited a broad coverage of the experimental structures, providing valuable insights into the functional aspects of lysozyme and CYP3A4. Furthermore, dpMDNM also covered lysozyme structures with relatively elevated energies corresponding to transient states not easily obtained by standard MD simulations, in conformity with nuclear magnetic resonance structural indications. This method offers an efficient and rational framework for comprehensive protein conformational sampling, contributing significantly to our understanding of protein dynamics and function.

## 1 Introduction

Proteins are nanoscale molecular machines that orchestrate essential cell functions, such as the enzymatic activity of metabolism,^1^ transport,^2^ storage of substances,^3^ cell defense^4^ and communication,^5^ gene expression and regulation,^6^ and cell architecture.^7^ X-ray crystallography studies have provided foundational knowledge about protein folding and structure–function relationships.^8,9^ Further, the development of solution experiments, such as atomic force microscopy (AFM),^10^ nuclear magnetic resonance (NMR) spectroscopy,^11^ X-ray free electron lasers,^12^ cryo-electron microscopy (cryo-EM),^13^ and single-molecule fluorescence^14^ have expanded our understanding of proteins as dynamic and flexible entities. They showed a wide range of motions enabling proteins to explore conformational landscapes that exhibit many low-energy wells corresponding to functionally relevant substates.^15^ These experiments have also revealed the importance of protein dynamics in their functions. Proteins’ motions are influenced by their specific folds, which have evolved through natural selection to enable proper functioning in biological environments, referred to as the structure–motion–function paradigm.^16^ Characterizing protein dynamics is now widely recognized as being valuable for gaining insights into their functions.

Molecular dynamics (MD) simulations are widely used to study protein conformational changes, being able to replicate, interpret, and guide experiments^17−20^ but can strive to describe large-amplitude or global conformational changes in large biomolecular systems, requiring the development of sophisticated enhanced sampling techniques.^21−24^ In this scenario, normal modes (NMs) are essential in efficiently capturing protein global conformational changes.^25−28^ Classical NM calculations are based on a physics-based forcefield and involve energy minimization and diagonalization of a Hessian matrix. Simplified approaches like elastic network models (ENMs),^29^ Gaussian network models,^30,31^ and anisotropic network model^32^ have also been proposed. Indeed, the low-frequency motions described by ENMs reproduce, to a large extent, those obtained in classical NM calculations. These findings strongly suggest that global protein motions arise primarily from their overall shape. Therefore, NMs provide an appropriate conformational space for exploring energetically relaxed structures and pathways.

Low-frequency NMs have been shown to correlate with dynamic modes of cytochrome P450cam (CYP101) measured by coherent neutron scattering, indicating the utility of NMs in capturing global motions.^33^ Several other studies have shown the effectiveness of NMs in describing conformational transitions observed in various experimental techniques, including small-angle X-ray scattering (SAXS),^34^ crystallographic structures,^35^ and cryo-EM.^36^ NMs have also been employed in molecular replacement in crystallography,^37^ identification of druggable regions for drug design,^38−40^ capturing relevant conformational changes in protein–protein recognition,^41^ exploring local loop conformations,^42^ and describing global motions in viral capsids,^43−45^ DNA,^46^ and RNA.^47^ These findings highlight the broad applicability and valuable insights provided by NMs in understanding protein and biomolecular dynamics across different systems and contexts.

Pioneering studies on NMs in proteins have focused on understanding the motions described by individual low-frequency NMs.^48−52^ Improved exploration of the conformational space was further achieved by combining low-frequency modes, resulting in better coverage of the subspace spanned by the experimental conformations. Transitions between apo and holo forms of 22 proteins^53^ were efficiently described by linear combinations of 20 lowest frequency NMs using a least squares fitting algorithm. Hybrid techniques that combine NMs and MD have been developed in further studies, such as the collective molecular dynamics method, which employs a Monte Carlo scheme to combine NMs and obtain energetically favorable paths between two known states.^54^ Later, another technique called Molecular Dynamics with excited Normal Modes (MDeNM)^24^ was proposed by our group, where multiple replicas are used to explore large conformational spaces under explicit solvent conditions by kinetically exciting linear combinations of NM vectors.^24,51^ Similar approaches were proposed by Bahar’s team, with the development of the ClustENMD technique,^55^ involving iterative cycles to generate pools of conformations displaced along combinations of NMs, followed by standard MD simulations from representative conformations. These techniques highlight the importance of carefully determining a set of NM combinations for effective protein conformational sampling.

MDeNM contains a root mean square deviation (RMSD) filtering function, preventing the generation of similar combined NM vectors for better conformational exploration.^24^ Kanada and collaborators have applied Bayesian optimization to explore the adaptive CG-ENM space efficiently, reducing the cost of finding suitable parameters to 10% of those performed by random sampling.^56^ Other researchers have developed software that allows NM linear combination for visual inspection, which is applied in studying tunnels and metabolite paths through the catalytic site of proteins.^57^ Altogether, these pieces of evidence show that an optimal strategy to produce and select linear combinations of NMs would be of great value in the context of enhanced sampling strategies. Further, there is still a need to understand how wholly and extensively the subspace described by low-frequency NMs is being explored. The present work addresses these points by presenting a minimization algorithm yielding an optimal distribution for the orientations of a set of NM combined vectors that generate protein conformations located on concentric hyperspheres to be submitted to standard MD for efficient protein conformational sampling. Our approach was applied to two proteins with different conformational behaviors: (i) hen egg-white lysozyme, where a dominant hinge-bending motion allows an open-to-close transition,^9,52,58^ and (ii) the human cytochrome 3A4 (CYP3A4), which is known to bind to various small molecules and shows high conformational plasticity.^59^ Instead of a unique dominant motion, several NMs are required to describe its conformational space adequately.

## 2 Methods

Consider an NM space of *D*-dimensions, where *D* is the number of a given system’s lowest frequency NMs. The system can be deformed in this space along a single or a combination of NMs. While a point in this space corresponds to a given conformation, a vector in the NM space can be understood as a driving direction to modify the conformation.^51,60^ From this perspective, we propose mapping the NM space into an Euclidean surface corresponding to a *D*-dimensional unit hypersphere. Each point on the hypersphere’s surface corresponds to a combination of NM vectors, which radiate from the center of the hypersphere, corresponding to the initial structure.

### 2.1 Uniform Positional Distribution on the Hyperspherical Surface

The energy function proposed by Kottwitz^61^ was utilized in a minimization procedure to disperse the points resulting from the intersection of a random distribution of vectors with the hypersphere. The purpose is to distribute these points evenly on the hypersphere’s surface. The energy function *E* is defined as
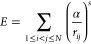
where α is a scaling factor that prevents the function from overflowing, *r*_*ij*_ is the Euclidean distance between points *i* and *j*, with *i* and *j* being integer numbers from 1 to *N*, and *N* being the number of points, with the condition that the points are constrained to remain on the hypersphere’s surface. The exponent *s* controls the inverse-power law, determining how the points become more separated as the energy *E* decreases. Ultimately, the energy reaches its theoretical global minimum when *s* approaches infinity (*s* → ∞). An explanation of the minimization process, including a flowchart (Figure S1), is provided in the Supporting Information.

**Figure 1:**
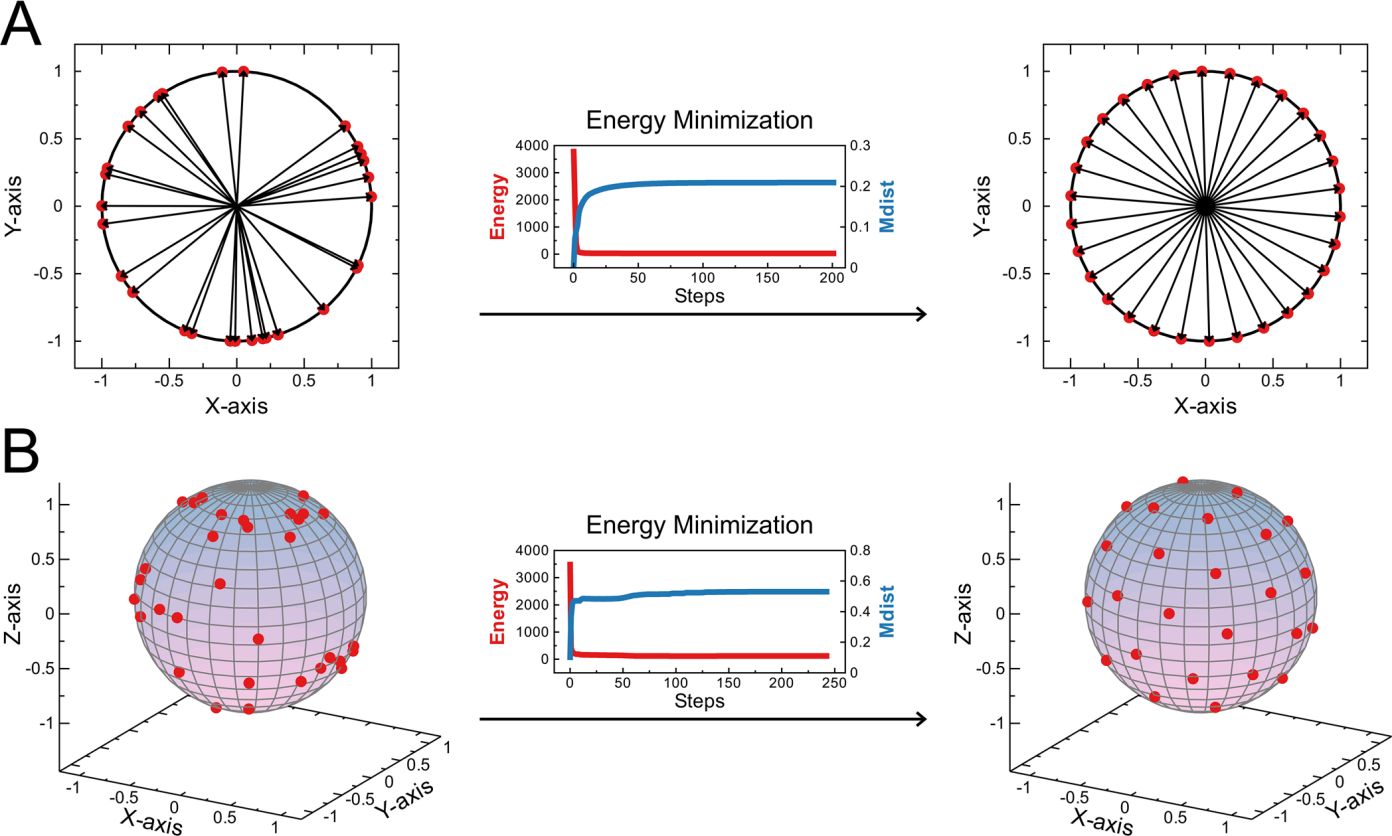
Obtaining a uniform distribution of points on the surface of an N-sphere using a minimization algorithm. Examples of (A) 2-dimensions (30 points in *X* and *Y* axes) and (B) 3-dimensions (48 points in *X*, *Y*, and *Z* axes) are shown, with the initial random distribution of points on the left and the final uniform distribution of points on the right. In the middle, the energy (red) and the minimal distance (Mdist, in blue) are shown in every step of the minimization process.

The process of the minimization algorithm can be visualized in both two- and three-dimensional spaces with several points distributed on the surface of a hypersphere (see Figure 1). In the bidimensional space, the hypersphere, which corresponds to a circle, was filled with 30 randomly distributed vectors/points (Figure 1A). The same was extended to a three-dimensional space, where the hypersphere corresponds to a sphere accommodating 48 vectors (Figure 1B). Initially, these vectors were randomly distributed (Figure 1A,B, left panel). Subsequently, the minimization algorithm was applied to achieve a regular distribution of points (Figure 1A,B, right panel). Throughout the minimization process, the decrease in energy (*E*) and the increase in the minimal distance between points (Mdist) can be observed (Figure 1A,B, middle panel), indicating the successful minimization and attainment of a uniform distribution of points.

### 2.2 Hypersphere and the Protein Normal Mode Space

**Figure 2:**
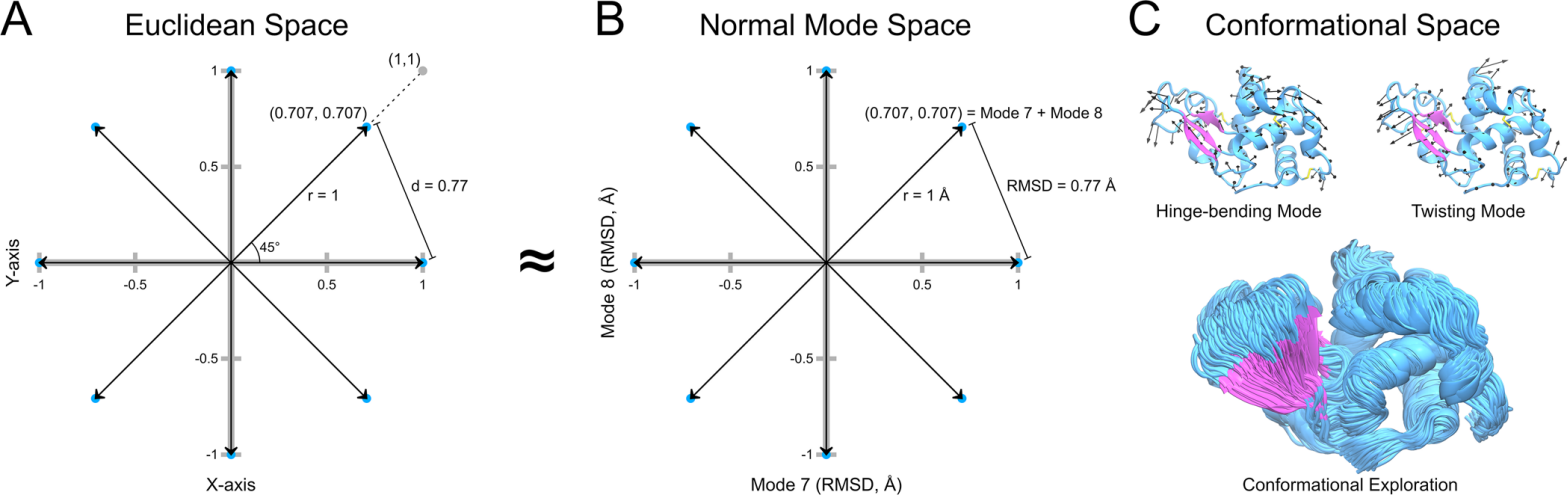
Relations between Euclidean space, normal mode space, and protein conformational space. The distribution of vectors in (A) Euclidean space (*X* and *Y* axes) is nearly identical to (B) NM space (modes 7 and 8), as illustrated by a set of eight vectors evenly distributed every 45° in the 2D space. The distance between the heads of adjacent pairs of vectors (*d*) and the length (*r*) of the vectors (normalized) in the Euclidean space are compared to those in the Cartesian space in terms of RMSD and the atomic displacement along the vector (1 Å) of the initial structure. Hinge-bending and twisting motions corresponding to modes 7 and 8 of lysozyme (C, upper panel) are combined for an efficient conformational exploration obtained in our simulations using the two modes and 30 vectors combining them (C, lower panel).

When navigating the NM space, selecting the vector set is crucial for efficiently exploring the protein’s conformational space (Figure 2). Figure 2A shows an example of eight vectors uniformly distributed in a bidimensional space. These vectors of unit length are evenly separated by 45°, resulting in an Euclidean distance *d* of 0.77. This arrangement is reflected in the NM space, where the distances between these points correspond to the RMSD between structures (Figure 2B). The center of the hypersphere (point at 0, 0) corresponds to the reference structure used in NM calculations, and NM vectors radiate from this point. For instance, in a bidimensional space, the lowest frequency internal NMs 7 and 8 can be considered generally, while mode 9 can constitute the third axis in three dimensions. Each NM vector was used to displace the protein by an RMSD of 1 Å, mapping the unit vector property in the NM hypersphere. The coordinates of each point on the hypersphere were used as weights for NM linear combinations, as described in a previous study.^24^ Therefore, the uniform distribution of points or vectors translates into motions facilitating a more extensive conformational exploration of the NM space (Figure 2C).

Distributing points on the surface of a hypersphere unveil intriguing mathematical properties. In a *D*-dimensional space, the kissing number represents the maximum count of nonoverlapping unit spheres distributed on a central unit sphere.^62,63^ While determining the kissing number for every dimension is a work in progress, solutions have been proposed for dimensions such as 1, 2, 3, 4, 8, and 24, consistently resulting in a symmetric distribution of points.^64,65^ These properties facilitate the application of the mirrored minimization algorithm developed in this study. Intriguingly, the kissing number produces a point distribution where the Euclidean distance to their neighbors is precisely 1, except for the third dimension, which exhibits a value of 1.05.^64^ Therefore, we focused on studying hyperspheres from two to eight dimensions, placing points according to their respective kissing numbers: 6, 12, 24, 40, 72, 126, and 240. In this context, the NM space was explored from two to eight dimensions, considering the lowest frequency NMs. The number of vectors for each case corresponded to their respective kissing numbers, aiming to obtain 1 Å displaced structures having a minimal RMSD of 1.00 or 1.05 Å between them.

### 2.3 Molecular Dynamics Simulation Protocol

In this work, we selected two proteins for the conformational study: hen egg-white lysozyme and human microsomal cytochrome P450 3A4 (CYP3A4). The crystallographic structures of lysozyme and CYP3A4 were obtained from the Protein Data Bank server (http://www.rcsb.org) under the PDB ID codes 3LZT and 1TQN, respectively. Water and small molecules were discarded, except for the heme group bound to CYP3A4.

The protonation states of residues in both proteins were calculated using the PROPKA webserver,^66^ with Arg105 considered neutral and Glu320 protonated in CYP3A4. The systems were constructed using CHARMM software version 41b1^67^ under the CHARMM36m force field.^68^ The patch PSUL was used to apply a bond between the sulfur atom of Cys442 and Fe^2+^ to stabilize the heme group. Given the focus of this work on studying large amplitude movements, each protein was placed in a rhombic dodecahedral box extending 20 Å from the farthest atom of the protein in *XYZ* axes. Subsequently, each system was solvated and neutralized with 150 mM NaCl. Equilibration and subsequent MD simulations were performed using the Langevin thermostat, implemented in NAMD version 2.13.^69^ Each system underwent an *NVT* ensemble simulation of 500 ps, using the Langevin piston, with a damping value of 1.0/ps and a time integration step of 0.1 fs. The non-hydrogen atoms of the protein were harmonically constrained with a force constant of 1.0 and 0.1 kcal/mol/Å^2^ applied to the backbone (including the heme group) and side-chain atoms, respectively.

Initially, each system was heated from 200 to 300 K, with a 1 K increment every 0.5 over 50 ps. The initial atomic velocities were generated according to a Maxwell–Boltzmann distribution, reassigned every 0.5 ps. The subsequent 450 ps were performed at 300 K, with a temperature coupling interval of 0.1 ps. An *NPT* ensemble was then applied using a Nosé–Hoover Langevin piston at a constant pressure of 1.013 bar with a time integration of 2 fs. Harmonically constrained atoms were maintained as previously described for 1000 ps. Harmonic constraints were gradually released in a subsequent *NPT* ensemble lasting 200 ps by decreasing the force constant by 0.1 kcal/mol/Å^2^ for the backbone (and heme group) and by 0.01 kcal/mol/Å^2^ for side-chain atoms every 20 ps until reaching zero. Finally, an unrestrained MD simulation of 300 ps was performed to accommodate the systems.

Nonbonded interactions between pairs of atoms within a 10 Å distance were calculated with a switching function between 10 and 12 Å, while long-range electrostatic calculations were treated with the particle-mesh Ewald method. Hydrogen bonds were constrained using the SHAKE algorithm.^70^ A harmonic force constant of 1.0 kcal/mol/Å^2^ was applied to restrain the center of mass of lysozyme or CYP3A4 near the center of the box. All of the simulations (standard MD, targeted MD (TMD), and MDeNM) with explicit solvents were carried out using NAMD. Each simulation started from the respective equilibrated systems.

### 2.4 Normal Mode Calculations

Before conducting NM calculations for lysozyme and CYP3A4 with its heme group included, each equilibrated structure underwent energy minimization in a vacuum with a distance-dependent dielectric constant of 2*r*. Initially, non-hydrogen atoms were harmonically constrained with a force constant of 1 and 0.1 kcal/mol/Å^2^ for backbone and side-chain atoms, respectively. Then, several minimization steps were performed by combining the steepest descent (SD) and conjugate gradient (CONJ) algorithms, progressively decreasing the constraints to zero. The final minimization utilized the adopted basis Newton–Raphson (ABNR) algorithm until an energy gradient of 10^–6^ kcal/mol/Å was achieved. The resulting minimized structures of lysozyme and CYP3A4 exhibited RMSDs with respect to their corresponding initial structures of 0.84 and 1.1 Å, respectively. After that, NMs with frequencies below 50 cm^–1^ were calculated using the diagonalization in a mixed basis (DIMB) method.^25,71^ NMs for each protein were considered according to the number of dimensions studied, from the lowest frequency NMs ranging from 7 to 14 (the first 6 NMs being nonrelevant rigid-body rotations and translations in *XYZ* directions).

### 2.5 Obtaining Restrained Structures along Normal Mode Vectors Followed by Free MD Simulations

**Figure 3:**
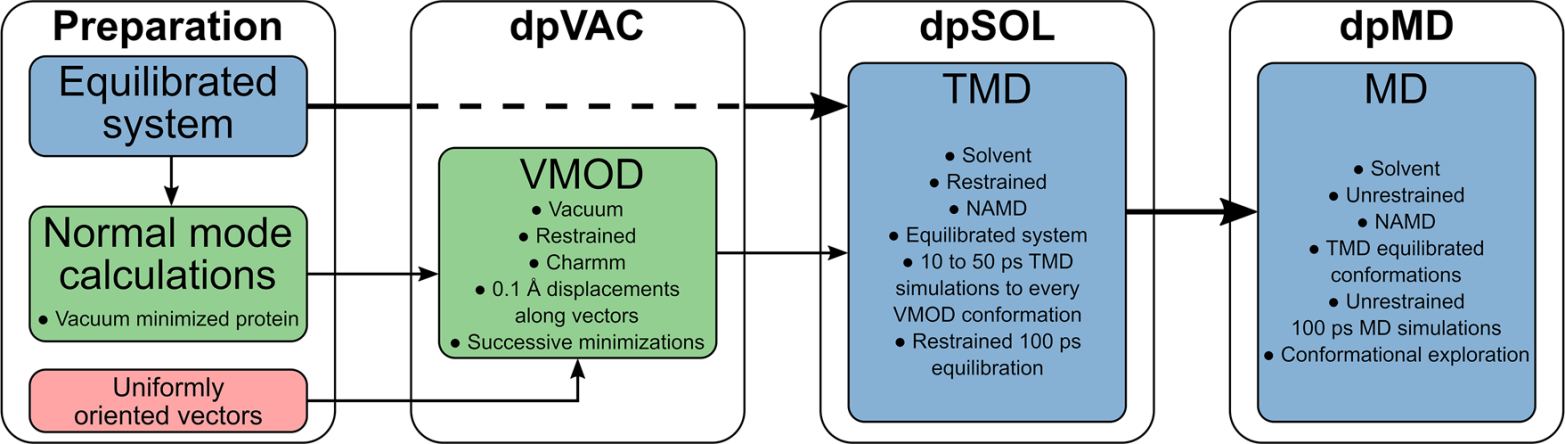
Flowchart outlining the dpMDNM approach, from system preparation to unrestrained MD simulation for efficient conformational exploration. Each box represents a step or a set of related steps, and arrows indicate the flow of the process. A preparation step is required to solvate and equilibrate the system properly. As explained in the article, the equilibrated protein is minimized in vacuum for NM calculations, while the minimization algorithm generates uniformly oriented vectors in the Euclidean space. In the first stage (dpVAC), the uniform set of vectors is mapped into the NM space, which is then used to displace the protein under harmonic restraints (VMOD module of CHARMM). The second stage (dpSOL) employs targeted MD (TMD) to generate all protein structures obtained by VMOD but now in a more realistic environment including solvent and ions, followed by a restrained short equilibration step. Finally, the third stage (dpMD) submits these final equilibrated structures to an unrestrained MD simulation for efficient conformational sampling.

To comprehensively and more thoroughly explore the NM space, we devised a novel approach termed “distributed points MD using NM” (dpMDNM) that consists of three stages. The first stage generates regularly spaced energy-minimized structures along the uniformly oriented vectors obtained by the hypersphere minimization algorithm (see the section above and Figure 1) in vacuum, termed dpVAC. The second stage performs targeted molecular dynamics (TMD)^72^ simulations for the solvated system taking the preceding generated protein structures as target points, termed dpSOL. These restrained structures are subsequently submitted to free MD simulations to explore further the conformational space termed dpMD (see Figure 3).

In more detail, a series of positional restraint potentials along the vectors were used for conducting the dpVAC stage with a method implemented in CHARMM termed VMOD.^73^ These structures are generated in a vacuum starting from the minimized structure used in NM calculations. VMOD allows the displacement of a given structure along an NM vector to desired distances from the reference structure by constraining them to a harmonic restraining potential,^74^ defined as
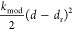
where *k*_mod_ is the force constant and *d*_r_ is the desired restraint distance along the given NM vector. *d* represents the current displacement from the initial structure calculated as a mass-weighted root mean square distance (MRMSD), as defined as
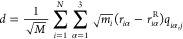
where *r*_*i*α_ is the coordinate of the atom *i* along one of the Cartesian axis α, *N* is the total number of atoms, *r*_*i*α_^R^ is the corresponding coordinate of the reference structure, *q*_*i*α,*j*_ is the corresponding element in the *j*th NM vector considered, *m*_*i*_ is the mass of the atom *i*, and *M* is the total mass. This equation corresponds to the scalar product between the MRMSD vector and the normalized NM vector. Successive minimization steps remove structural distortions and achieve energetically relaxed conformations.^73^ For this stage, CHARMM was used. In the dpSOL stage, TMD simulations are carried out for a solvated system, starting from the equilibrated structure and driving it toward the successive structures obtained in the preceding stage. In the dpMD stage, the new set of generated solvated conformations was further submitted to standard MD simulations to obtain an equilibrated, unbiased conformational ensemble. These last two stages were performed with NAMD. The novelty of the dpMDNM approach consists of conducting extensive protein conformational exploration by generating uniformly distributed conformations along NM vectors and their combinations under solvated conditions before unrestrained MD simulations.

These three simulation stages were applied to lysozyme and CYP3A4. Initially, conformations of each protein were generated in vacuum along the uniformly oriented combined NM vectors using VMOD. This consisted of 30 displacements considering all protein atoms, with MRMSD steps of 0.1 Å, progressing until 3 Å was reached along each vector. For lysozyme and CYP3A4, the positional harmonic potential force constant (*k*_mod_) was set at 10^4^ and 10^6^ kcal/mol/Å^2^, respectively. A combination of SD and ABNR algorithms was employed during the minimization step, consisting of 10,000 and 20,000 steps with a soft tolerance gradient of 0.1 and 0.01 kcal/mol/Å, respectively. Subsequently, TMD was utilized for the equilibrated solvated system to displace it to their target conformations obtained from VMOD using an elastic constant of 5000 kcal/mol/Å^2^ for 10 or 50 ps, respectively. Subsequently, the generated conformers were equilibrated for 100 ps using position restraints first on solute atoms to accommodate the solvent and after that to an additional 100 ps free standard MD with frames collected every 5 ps.

### 2.6 Molecular Dynamics with Excited Normal Modes (MDeNM)

To evaluate the effectiveness of dpMDNM in exploring the conformational space, we conducted a comparison with a previously developed method termed “MD with excited NMs” (MDeNM),^24,51^ in the specific case of lysozyme. MDeNM is a multireplica method to explore protein conformational changes through successive kinetic excitations along a diverse set of combined NM vectors using short MD simulations.^24,51^ A replica consists of kinetic excitation cycles along a combined NM vector, displacing the system by larger distances. The additional energy along a single degree of freedom rapidly dissipates to other degrees. It also decreases due to the thermostat activity during the simulation before another excitation is applied.

To explore the NM space of lysozyme, a bidimensional set of 30 optimally distributed vectors was generated using the hypersphere minimization algorithm previously described (Figure 1) considering modes 7 and 8. This set of vectors was employed for dpMDNM and MDeNM. With dpMDNM, we generated 30 points for each of the 30 vectors, whereas 10 excitation cycles followed by 5 ps relaxation after each excitation were performed with MDeNM. The additional temperature added in the NM space was set to 5 K. Finally, 500 ps of standard MD were carried out from each structure generated by dpMDNM and MDeNM, with frames collected every 5 ps.

Initially, MDeNM was devised to run in CHARMM using an appropriate script.^24,51,75^ However, we implemented the same methodological procedure for running with NAMD through Python programming for optimized performance.^76^

### 2.7 Structural Analysis

To comprehensively assess the conformational samplings obtained with dpMDNM and MDeNM for lysozyme and CYP3A4, we compared them with the conformational space covered by the existing sets of X-ray and NMR structures.

For lysozyme, the following X-ray PDB structures were selected: 3B6L, 5JEN, 193L, 1HEO, 2IFF, 1IR8, 1IR7, 1IR9, 1HEM, 1H6M, 1KXW, 1IOT, 3A3Q, 1HER, 1HEN, 1LSG, 1FLY, 1FLW, 1FLQ, 1FN5, 1FLU, 1LZD, 1KXY, 3OJP, 4YEO, 1NBY, 1NBZ, 1HEQ, 5HMV, 1LZG, 1V7S, 1IOS, 1NDG, 3OK0, 1HEP, 3WVX, 1A2Y, 1IOR, 6P4A, 1AT6, 1LSN, 1KXX, 1UID, 1IOQ, 1UIC, 1BQL, 3WW5, 3WW6, 6F1L, and 3QY4. Additionally, NMR PDBs 1E8L and 1IVM were included. For CYP3A4, X-ray structures were selected based on the three states of the proteins:^77^ C-state PDBs: 1TQN, 1W0E, 4I3Q, 1W0F, 5A1R, 5A1P, 1W0G, 3UA1, 4K9V, 4D75, 4D6Z, and 4NY4; O1-state PDBs: 3NXU, 4I4G, 4I4H, 3TJS, 4K9W, 4K9X, 4D78, and 4D7D; and O2-state PDBs: 4K9T, 4K9U, 2V0M, and 2J0D. Any missing atoms in the experimental structures were modeled using the Swiss-model webserver,^78^ defining their respective crystallographic structures as templates.

To calculate the volume of Channel 2^77^ for all CYP3A4 structures, we employed Epock.^79^ Two spheres were positioned along the channel: one at the entrance, near the F′G′ region (formed by a residue range from Pro218 to Leu236), with a radius of 12 Å, and another, deeper in the channel, close to the heme group, with a radius of 10 Å. Each sphere had a smaller sphere at its center half the size, defined as a contiguous seed region. We determined a grid spacing of 0.5 Å with a contiguous cutoff equal to 2 Å. The spatial arrangement of each sphere in CYP3A4 is illustrated in Figure S2A. Additionally, the Orientations of Proteins in Membranes (OPM) database was used to predict the orientation of CYP3A4 in a flat DOPC (1,2-dioleoyl-*sn*-glycero-3-phosphocholine) bilayer, considering membrane penetration depth and transfer energy (Δ*G*) values.^80^

The calculation of the breathing angle of lysozyme related to the hinge-bending motion followed the methodology outlined in previous work,^81^ with slight modifications in atom selection. The center of mass of three groups of Cα atoms was defined as follows: (i) the range of residues Trp28–Ala31 and Ala110–Asn113; (ii) Thr89–Val92; and (iii) Trp43–Arg45 and Trp51–Tyr53 (Figure S2B). To determine the distance from the F′G′ region to the β1 region of CYP3A4, the center of mass of Cα atoms of Pro218–Leu236 and Val71–Ile84 were selected, respectively (Figure S2C). The relative secondary structure (SS) content and the variation in the solvent-accessible surface area (SASA) were calculated as ratios to the values corresponding to the equilibrated structure of each protein serving as a reference. For SASA calculations, a probe radius of 1.4 Å was utilized. The root mean square fluctuation (RMSF) calculation was performed considering the Cα atoms of proteins. All structural measurements, including the breathing angle and distance calculations, were performed using CHARMM scripting.^67^ Visualization of structures was realized with VMD,^82^ and graphical representations of data were generated using gnuplot v.5.2 (http://www.gnuplot.info).

## 3 Results and Discussion

The challenge of obtaining uniform point distributions on the surface of a sphere initially emerged in geometry but found rapid applications across diverse fields such as mathematics, chemistry, physics, biology,^64^ geosciences,^83^ materials science,^84^ and crystallography.^64^ Current geometric methods designed for three dimensions are often based on a spiral distribution^85^ or an octahedral sphere partition.^83^ Methods utilizing spherical volume partition^86^ and zonal areas partition^87^ have been developed for higher dimensions. In addition, minimization algorithms have been effective in three^61^ and higher dimensions.^88^ In this study, we employed such a minimization algorithm to achieve a uniform distribution of points on the surface of a hypersphere. Subsequently, this method was utilized to explore the NM space of lysozyme and CYP3A4.

### 3.1 Finding a Uniform Distribution of Points on a Hypersphere

The implemented minimization algorithm in this study was initially tested to generate a uniformly distributed set of points in two and three dimensions, using 30 and 48 points, respectively. In both cases, the global minimum was reached with an energy gradient tolerance of 0.001 and 10 minimization cycles (refer to Supporting Information). The minimal distance between pairs of points (Mdist) was gradually increased, reaching final values of 0.12 (Figure 1A) and 0.53 (Figure 1B) for two and three dimensions, respectively (refer to Videos V1 and V2). Additional minimization algorithm tests using different points also reproduced the minimal distances for three dimensions found by Kottwitz^61^ (Table S1).

Our interest extended to obtaining distributions according to the kissing numbers and achieving Mdist values as close as possible to 1^64,65^ (see Table 1). Merely 5% of 100 minimization runs in four dimensions resulted in successful distributions (Mdist = 1), considering a kissing number of 24 points (Table 1). This outcome suggests that the energy space contains numerous bottlenecks, making it challenging to reach a global minimum. To overcome this limitation, we implemented the mirrored minimization algorithm (see Methods), enabling the discovery of a uniform distribution of points in every run. This approach also facilitated a uniform distribution of points in higher dimensions up to 8 (refer to Supporting Information).

**Table 1:** Comparison between the Hyperspherical and Normal Mode Spaces

dimensions/normal modes	points/vectors	neighbors	Mdist	min. RMSD	
				lysozyme	CYP3A4
2	6	2	1.000	0.999	1.000
3	12	5	1.051	1.049	1.051
4	24	8	1.000	0.998	1.000
5	40	12	1.000	0.998	1.000
6	72	20	1.000	0.998	1.000
7	126	32	1.000	0.998	1.000
8	240	56	1.000	0.998	1.000

### 3.2 Toward a Comprehensive Exploration of Normal Mode Spaces

The vectors uniformly distributed within the hyperspherical space can be regarded as NM vectors (Figure 2). We correlated the Mdist values of the hyperspherical space with the minimal RMSD values in the real space, resulting in nearly identical values across every dimension studied (Table S2). The difference between both measures consistently remained below 0.001 for every case, with a robust Pearson correlation (*R* > 0.999) observed in every set of combined NMs for lysozyme and CYP3A4 (Table S2). The similarities between these two spaces allow for the definition of a uniform set of NM vectors derived from the minimization of points on the surface of the hypersphere (Figure 2). In addition, as the number of neighbors increases with the consideration of more dimensions, filling a larger space while preserving a given minimal distance necessitates more points or vectors. This is crucial when aiming to populate a space composed of several NMs for comprehensive protein conformational sampling of the NM space.

Exploring multidimensional NM space presents a formidable challenge. To illustrate, adhering to a target separation of 1 Å between NM vector’s end points could yield a staggering 196.560 vectors in a 24-dimensional space.^65^ Previous studies have attempted conformational sampling by employing combinations of modes as favored directions, but they often fell short of ensuring uniform exploration. For instance, a study on myotoxin using 8 NMs incorporated only around 50 vectors with minimal distances exceeding 1 Å,^89^ representing only 20% of the anticipated 240 vectors for such a case (Table 1). In another study, 11 NMs were combined to produce 62 vectors,^90^ equivalent to just 10% of the 582 vectors expected in that dimensionality.^65^ Furthermore, Lima and co-workers investigated the conformational space of the human prion protein by combining 74 NMs but using only 2000 vectors.^91^ These studies underscore evident limitations in the existing protocols employed for fully exploring NM spaces.

### 3.3 Uniform Exploration of the Protein Normal Mode Space

We subsequently assessed the conformational exploration using uniformly oriented NM vectors with the dpMDNM approach. Seven explorations with different dimensionalities and different NM combinations were performed for each protein. The NMs considered are the lowest frequency ones that describe large-scale internal motions ranging from 7 to 14.

**Figure 4:**
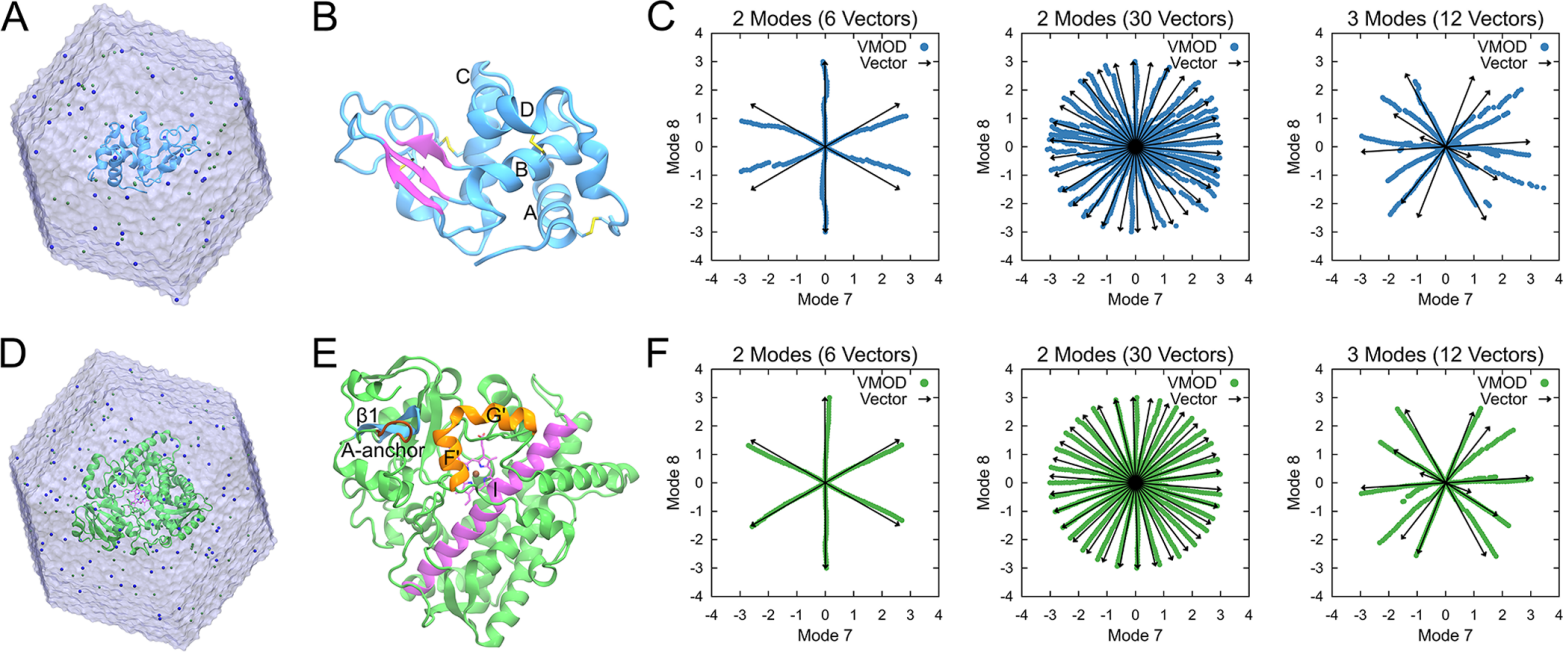
Lysozyme and CYP3A4 were used as models for conformational sampling. Each protein was placed in a rhombic dodecahedron box (transparent surface) with (A) lysozyme and (D) CYP3A4, shown as cyan and green cartoons, respectively. Na^+^ and Cl^–^ are shown as blue and green spheres, respectively. Structural details of (B) lysozyme with its helices A to D, β-sheet (purple cartoon), and disulfide bonds (sticks, with sulfur shown as yellow) and (E) CYP3A4, with helices I (purple), F′ and G′ (orange), β1 (cyan), and A-anchor (brown) highlighted. The heme group and Fe^2+^ are shown as purple sticks and brown spheres, respectively. Structural displacements performed by VMOD (colored circles) along combined NM vectors (black arrows) considering different numbers of modes and different numbers of vectors for (C) lysozyme and (F) CYP3A4.

The first stage of the conformational exploration (see Figure 3) was to create displaced structures in the NM space in two or three dimensions for lysozyme and CYP3A4, as shown in Figure 4, where arrows denote the NM combined vector directions. Colored points correspond to displaced structures in steps of 0.1 up to a limit of 3 Å using harmonically restrained energy minimizations in a vacuum (as explained previously). It was observed that lysozyme generated structures consistently adhered to NM vectors up to 1 Å displacements, after which increasing deviations occurred until reaching 3 Å in several directions (see Figure 4C), even when using a strong restraint potential with VMOD. The deviation above 1 Å might signify high energy and unfavorable conformation, as evidenced by an NMR unbound-state ensemble, which exhibited an average RMSD of 1.50 Å compared to the crystal structure.^92^ This is consistent with the backbone RMSD values of the experimental ensembles considered in our work (Figure S3A). Conversely, displacements on CYP3A4 demonstrated a remarkable adherence of structures to NM vectors even at displacements up to 3 Å (Figure 4F), suggesting a larger accessible conformational space with fewer energetic restrictions. This observation aligns with the known structural plasticity of CYP3A4 reported in the literature.^93,94^ Indeed, the experimental ensemble used in our work showed conserved CYP3A4 folding with flexible loop regions, presenting average backbone RMSD values below 2 Å (Figure S3B). The second and third stages (Figure 3) used the precedent sets of structures to generate ensembles with the dpSOL and dpMD simulations in solvent (see Methods). The equilibrated structures obtained from standard MD were then compared with crystallographic or NMR experimental structures. The results obtained for both proteins are presented in the following chapters.

#### 3.3.1 Lysozyme: Dynamics Is Governed by a Dominant Hinge-Bending Motion

Hen-egg white lysozyme is a globular protein composed of 129 amino acid residues.^9^ Its native tertiary structure contains four disulfide bonds and is formed by two domains: the α-domain, consisting of four α-helices and one 3_10_-helix, and the β-domain, which contains a β-sheet structure composed of three antiparallel strands and one 3_10_-helix^58,95^ (Figure 4A,B). Lysozyme catalyzes the hydrolysis of β-1,4 glycosidic linkages of cell wall polysaccharides that bind in the cleft between α- and β-domains,^58^ which acts as a hinge during the catalytic process, underlining the dynamic nature of lysozyme’s function and structural transitions.

Lysozyme is a typical protein used in structural and functional studies to validate and refine various methodologies, such as normal mode analysis,^52^ X-ray crystallography,^96^ NMR,^81,97^ small-angle neutron scattering,^98^ cryo-EM,^99^ AFM,^100^ and high-pressure techniques aimed at modulating enzyme activity.^101^ Therefore, the abundance of data available for lysozyme, resulting from its extensive use as a benchmark in diverse experimental setups, places it as an invaluable test case for validating theoretical approaches. For comparisons, we selected 50 crystallographic structures exhibiting high sequence identity and NMR ensembles corresponding to both unbound (PDB ID: 1E8L)^92^ and bound states (PDB ID: 1IVM)^102^ (refer to Methods for the pdb list). Lysozyme experimental ensembles show a rigid region in the α-domain and flexible residues in the extremity of the β-domain that allow its hinge-bending motion (Figure S4).

#### 3.3.2 dpMDNM Covers Lysozyme Experimental Conformations

**Figure 5:**
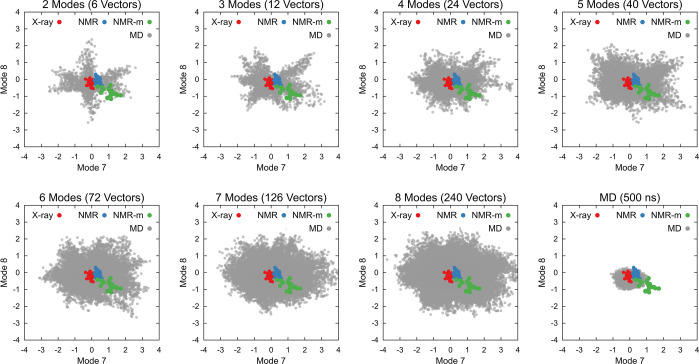
Lysozyme conformational sampling using dpMDNM. All seven ensembles of conformations generated using different numbers of modes and vectors, and that from standard MD, are shown as gray circles. X-ray, free-state, and bound-state NMR conformations are shown as red, blue, and green circles, respectively. The conformations were mapped onto a bidimensional space, spanned by modes 7 and 8.

Figure 5 illustrates the conformational exploration produced by dpMDNM and a 500 ns standard MD. Precisely, only unrestrained structures generated in the third stage (dpMD) are plotted. With an increment in the number of combined modes, more vectors are considered while consistently maintaining the minimal distance (Mdist) at 1 Å (Table 1). The exploration that uses only the first two modes required only six vectors, resulting in unvisited regions between them. These gaps in the conformational space were more effectively filled when linearly combining four or more NMs. Significantly, in all cases, the exploration facilitated the sampling of experimental structures obtained through X-ray crystallography and NMR. Conversely, structures from 500 ns of standard MD resulted in a restricted sampling around the unbound conformations.

**Figure 6:**
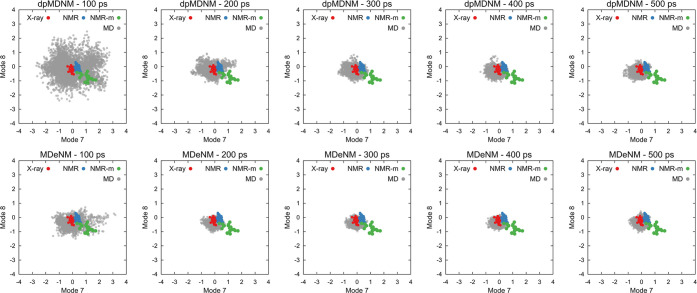
Temporal conformational exploration of dpMDNM and MDeNM during 500 ps of unrestrained MD simulations. Conformations from 30 vectors combining modes 7 and 8 are displayed at every 100 ps as gray circles. X-ray, free-state, and bound-state NMR conformations are shown as red, blue, and green circles, respectively.

Previous studies have suggested that lysozyme dynamics is primarily dominated by two low-frequency NMs: one related to hinge-bending and the other to twisting movements of its domains.^24,52^ To explore these features of lysozyme, an additional set of 30 vectors to the initial six vectors combining modes 7 and 8, was considered to generate a wider exploration with 500 ps of dpMDNM. The projection of structures onto the plan of modes 7 and 8 was displayed for every 100 ps in Figure 6.

We also achieved 500 ps MDeNM simulations for comparative analysis. Both approaches effectively sampled the entire space spanned by the experimental structures. However, during the first 100 ps, dpMDNM covered a broader area. By 200 ps, the area sampled with dpMDNM became almost comparable to that achieved with 100 ps of MDeNM. This observation suggests that lysozyme structures in dpMDNM simulations progressively relaxed toward energetically favored regions, which MDeNM already delimited. By 500 ps, both approaches converged to a similar conformational distribution, encompassing unbound structures (Figure 6), similar to that observed in standard MD (Figure 5). Notably, the poor sampling of NMR bound-states at 200 ps and beyond can be attributed to the likelihood that such open structures are induced by interactions with the substrate.^102^ Consequently, relatively higher energy structures of the apo native lysozyme must be effectively stabilized only when the substrate is present. These structures are well sampled in short 100 ps simulations of dpMDNM or MDeNM. The sequence of structural projections onto the NM space as a function of time looks like a convenient way to detect them.

**Figure 7:**
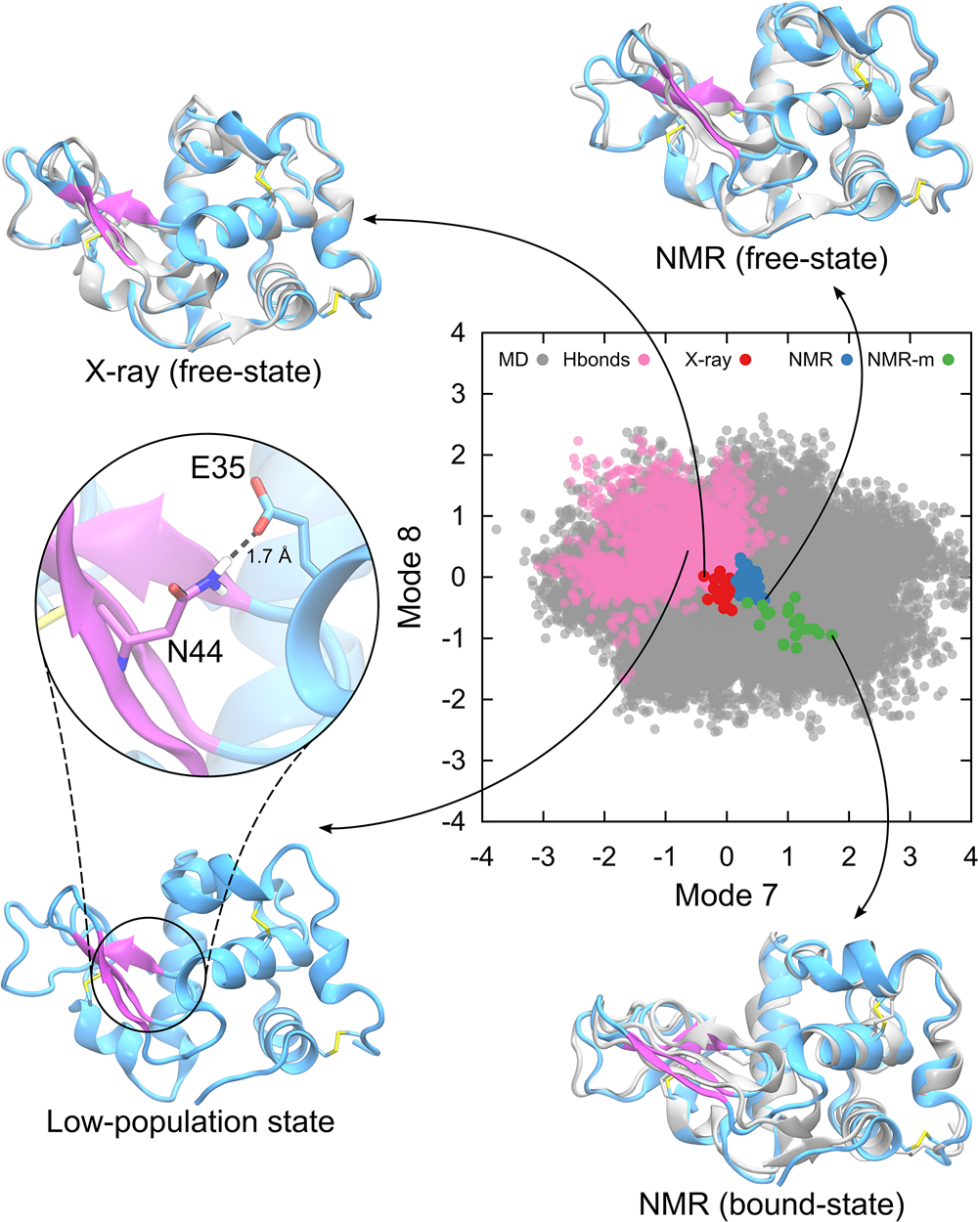
Lysozyme structural features of dpMDNM sampled conformations using 8 NMs that were uniformly combined. They are depicted in gray, X-ray structures in red, NMR free-state conformations in blue, and NMR bound-state in green, represented as circles. The structural similarities between experimental (gray) and theoretical conformations (cyan and purple) are shown as cartoons after superposition. A low-population state characterized by a salt bridge between residues E35 and N44 is highlighted in pink circles.

NMR experiments have also identified a low-population intermediate state of lysozyme associated with substrate release, characterized by a hydrogen bond between Glu35 and Asn44 side chains.^97^ The occupancy of this hydrogen bond was quantified to determine the presence of this distinct conformational state. The dpMDNM approach successfully sampled them (appearing as pink points in Figure 7), whereas neither MDeNM nor the 500 ns MD was sufficient.

To elucidate crucial structural aspects of lysozyme, we reported the generated structures into a bidimensional space defined by the breathing angle (θ)^81^ and the radius of gyration (*R*_g_). This facilitated a comprehensive comparison of all conformations produced in the present study with experimental ones (refer to Figure S5). Strikingly, we observed a consistent trend, with dpMDNM exhibiting more extensive space sampling than 500 ns MD simulations. It is worth mentioning that unrestrained 500 ns simulations did not explore the bound state (Figures 5 and S5, green points). Our results demonstrate that dpMDNM successfully explored all lysozyme conformations identified experimentally in the NM space (Figure 7) and the structural space (Figure S5).

#### 3.3.3 CYP3A4: A Promiscuous Protein

Cytochrome P450 (CYP) constitutes a vast superfamily of heme-containing enzymes encompassing more than 300,000 sequences identified in bacteria, fungi, animals, and plants.^103^ These enzymes play pivotal roles in numerous biosynthetic pathways, metabolizing over 90% of all chemicals, accounting for the main enzyme processing approximately 13% of chemicals and 27% of drugs.^59,104^ The remarkable plasticity exhibited by CYP enzymes in binding and processing diverse molecules positions them as potential targets for protein engineering and applications,^105^ underscoring the intriguing structural and functional dimensions of CYP enzymes in the intricate landscape of molecular processing.

CYP enzymes present a conserved global fold characterized by the arrangement of twelve α-helices (A to L) forming the α-domain, with some helices surrounding the heme group, located in the active site, and by five β-sheets (β1–5), the majority of which are organized in an associated region known as the β-domain^106^ (Figure 4D,E). In certain CYP subgroups, extended helical segments are observed, denoted by the corresponding helix letter with an added quotation mark, such as the F′ and G′ helices^93^ (Figure 4E). These two extended helices, together with a loop region called A-anchor, constitute a specific region related to membrane insertion and to the entrance of a cavity that drives compounds into the active site^107^ (Figure 4E). An X-ray study has described three major conformations of CYP3A4 concerning the orientation of its FF′ loop, a region modulated by ligands, resulting in three distinct conformational states: C-state, O1-state, and O2-state.^77^ These states were duly considered in our analysis.

#### 3.3.4 Crystal Structures Do Not Describe CYP3A4 Global Motions

**Figure 8:**
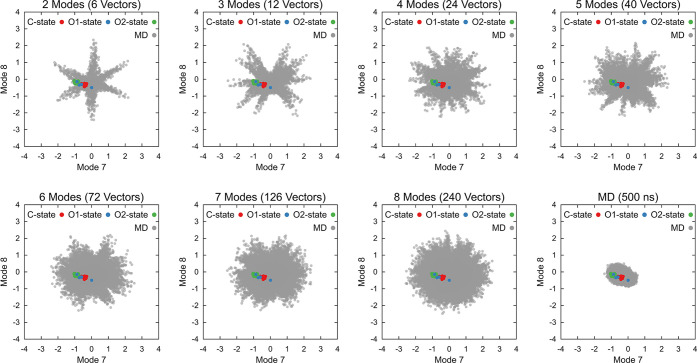
Conformational sampling of CYP3A4 using dpMDNM. All seven generated ensembles of conformations ranging from 2 to 8 NM dimensions mapped into the space of modes 7 and 8, and the ensemble from standard MD, are depicted as gray circles. The X-ray structures from C-, O1-, and O2-states are indicated by red, blue, and green circles, respectively.

The conformational exploration of CYP3A4 followed a strategy similar to that adopted for lysozyme, resulting in seven ensembles of conformations (see Methods). These ensembles were analyzed based on their distribution in the NM space. They were subsequently compared to C-, O1-, and O2-states as defined in the study by Benkaidali et al.^77^ Once again, it was evident that broader explorations of the NM space occurred when four or more modes were combined, while structures derived from standard MD simulations were confined to the proximity of the reference structure (refer to Figure 8). Crucially, all distinct states of CYP3A4, as elucidated in X-ray structures, were encompassed in dpMDNM and standard MD ensembles when plotted in the NM space. It is noteworthy, however, that most of these X-ray states are superposed in the NM space (Figure 8), rendering them challenging to distinguish solely through the lowest frequency NM projections.

Conformational changes of CYP enzymes play a role in facilitating a multistep binding process into their active site.^94,108−110^ This intricate mechanism is exemplified by the classic case of testosterone.^111^ Fluorescence resonance energy transfer (FRET) experiments have further validated a peripheral site near the F′ and G′ helices.^112^ Moreover, studies using NMs have identified robust dynamic similarities in CYP proteins, characterized by conserved global movements associated with their functional activity, highlighted by a flexible region encompassing the F and G helices.^113^ Neutron scattering measurements of CYP101 have provided insights into an NM associated with tunnel opening caused by motions of F and G helices, leading to the exposure of its active site.^33^ Further investigations have identified a region around the F′ and G′ helices as the primary cavity for substrate uptake in CYP enzymes into the active site, referred to as Channel 2.^114^ The surrounding residues in this region exhibit significant diversity, contributing to substrate selectivity.^115^ dpMDNM efficiently captured these global motions, as discussed in detail below.

#### 3.3.5 dpMDNM Explores the Channel 2 Dynamics of CYP3A4

**Figure 9:**
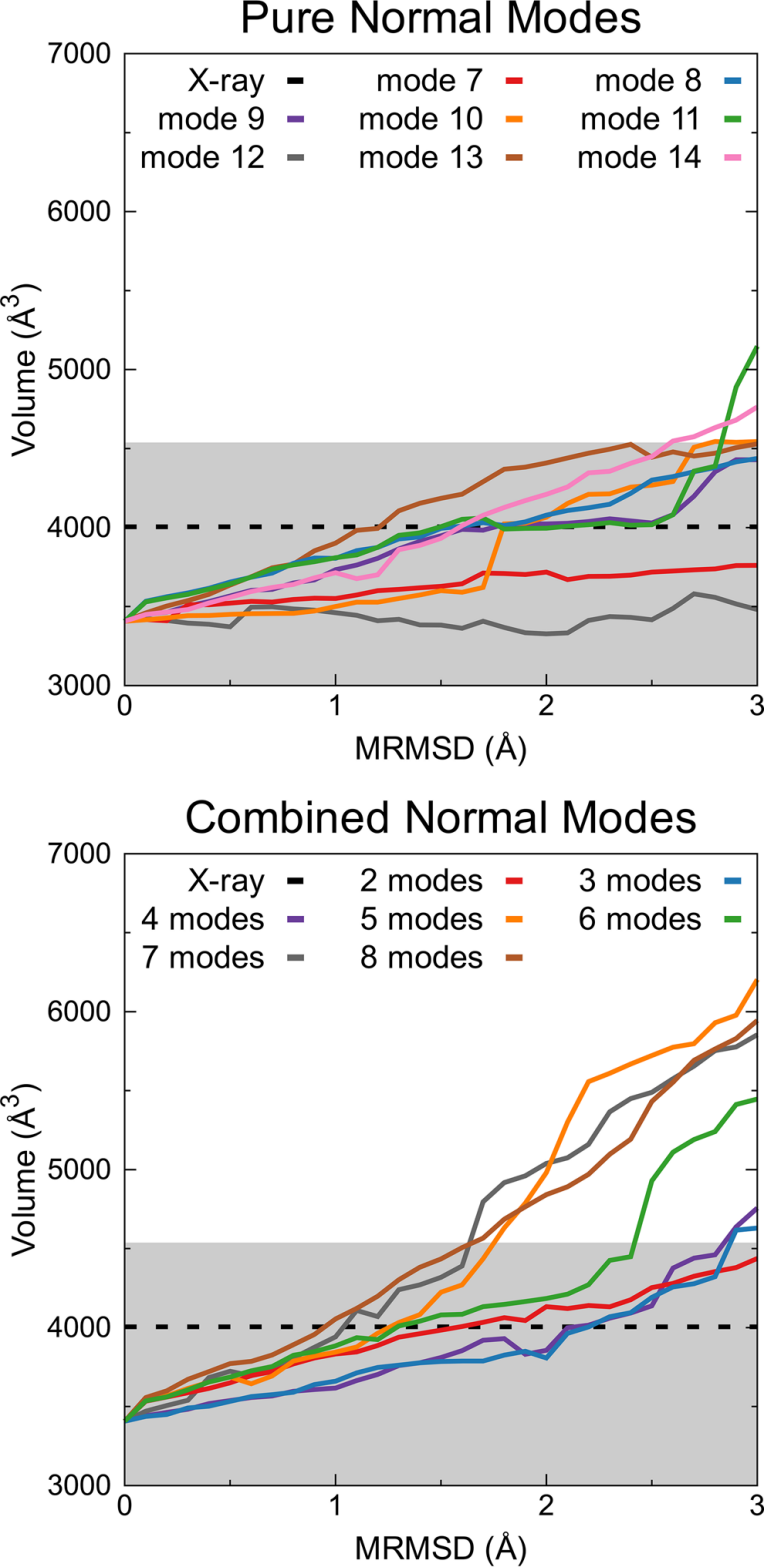
Dynamics of Channel 2 volume as a function of structural displacement using VMOD along individual (upper panel) and combined NMs (lower panel). The X-ray structure corresponds to the black dashed line, and the volume range obtained from standard MD is gray.

Analysis of conformational changes in Channel 2 was conducted through volume measurements using Epock^79^ (refer to Figure 9). The dynamics of such a channel are visually depicted in Figure 9, where the X-ray structure initially exhibits a volume of 4005.00 Å^3^ (Figure 9, dashed black line). Throughout 500 ns of standard MD simulations, the channel’s volume fluctuates between 2449.50 and 4538.12 Å^3^ (Figure 9, gray region). In both cases, the initial structure corresponds to the energy-minimized structure of CYP3A4 used for the NM calculation, exhibiting a reduced volume of 3407.12 Å^3^ (Figure 9, starting point in both plots). This constriction is likely due to vacuum conditions during the exhaustive minimization steps. Additionally, individual NMs from 7 to 14 were considered to identify the ones responsible for opening Channel 2 (Figure 9, upper panel). Such an analysis reveals that all modes, except for 7 and 12, increase the volume cavity of Channel 2. Mode 11 induces the maximum cavity volume to 5147.75 Å^3^. The atomic displacements of mode 11, as observed in Figure S6A, encompass motions previously identified experimentally in CYP101.^33^ Furthermore, several vectors from NM combinations increase the volume cavity of Channel 2 to values surpassing those observed in standard MD simulations, notably after including mode 11 (Figure 9, lower panel). Figure S6B,C showcases structures with the largest volumes obtained from standard MD and dpMDNM, respectively. Notably, dpMDNM captures a pronounced outward movement of F′ and G′ helices. These findings underscore the significance of combining NMs to generate diverse deformation patterns, facilitating a comprehensive conformational exploration of CYP3A4.

The determination of the volume cavity of Channel 2 and the channel opening (measured as the distance between the F′ and G′ helices and β-1; see Methods) served as structural reaction coordinates for the analysis of CYP3A4’s conformational exploration across all ensembles generated by dpMDNM and standard MD simulations (refer to Figure S7). Conformational exploration using only two NMs was notably restricted, aligning with the displaced structures generated by combined NM vectors in that scenario (Figure 8). However, as three or more modes were combined, the conformational exploration expanded, effectively encompassing the experimental structures (Figure S7). Once again, all three conformational states described in the X-ray structures were grouped, mirroring the observation in the NM space (Figure 8). This suggests that these crystallographic structures may represent the final ligand-bound conformations of CYP3A4.^94,110,116^ Conversely, several studies propose that intermediate conformations of CYP enzymes are related to changes in regions around the F′ and G′ helices and further opening of the active site.^33,111,117−119^ Notably, these intermediate conformations were effectively covered by both dpMDNM and standard MD simulations (Figures 9 and S7). In a final analysis, the structures with the biggest volume from each ensemble generated by the third dpMDNM stage (dpMD) were selected to assess their anchoring ability on membranes using the OPM server.^80^ Surprisingly, these conformations exhibited more favorable binding energies than the crystallographic structure (Figure S8), suggesting that the exposure of A-anchor and F′G′ helices facilitates membrane-anchoring of CYP3A4, aligning with existing literature.

#### 3.3.6 dpMDNM Enhances the Understanding of CYP3A4’s Function

The conformational exploration of CYP3A4 conducted in this study revealed motions crucial to the enzyme’s functional aspects, including movements of the F′ and G′ helices, Channel 2 volume dynamics, exposure of the active site (as illustrated in Figures 9 and S7), and the emergence of energetically favorable, membrane-oriented conformations (Figure S8). These conformations are directly linked to its function.^33,111,117−119^ They might serve as a source of intermediate structures, providing valuable insights for researchers seeking a comprehensive understanding of the enzyme’s multistep binding process,^94,110^ as well as its promiscuity in processing a diverse array of molecules, which is of great interest to the pharmaceutical market^59,104,116^ and protein engineering.^105^ The structural insights obtained through dpMDNM in the case of CYP3A4 hold significant value for understanding the functioning of other CYP enzymes. This is particularly important in light of the highly conserved global movements observed in CYP proteins.^113^ The findings of this study provide a window into the functional regions and common features shared across the broader CYP enzyme family.

### 3.4 dpMDNM Facilitates Extensive Conformational Sampling While Preserving the Structural Integrity of Proteins

In this study, we conducted a comprehensive conformational exploration of lysozyme and CYP3A4, utilizing uniformly oriented combined NM vectors. In contrast to prior works,^24,89−91,120,121^ we expanded our exploration by generating a higher number of vectors, encompassing combinations until eight NMs (7 to 14). A recent study suggests that a robust conformational description can be achieved by considering the four to five lowest frequency NMs.^23^ Indeed, our results demonstrate satisfactory conformational exploration, particularly when combining four or more lowest frequency NMs. Notably, the exploration expands further when incorporating eight modes, as evident in Figures 5 and 8. We conducted further structural analysis to ensure the quality of these diverse conformations in our results.

**Figure 10:**
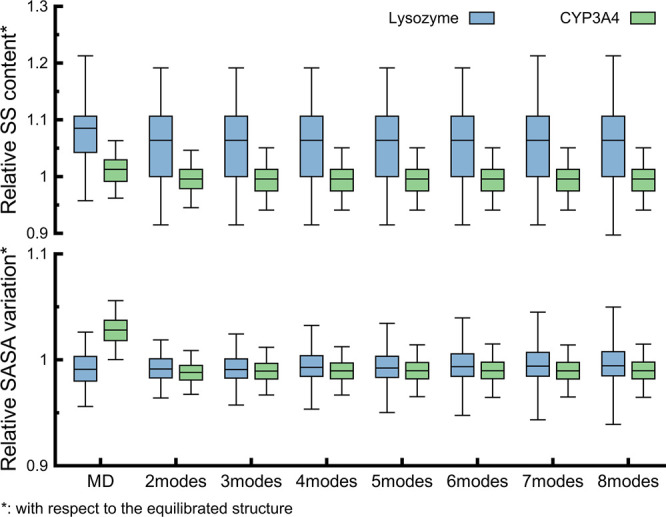
Structural quality of conformations obtained from standard MD and dpMDNM simulations. Relative secondary structure (SS) content and relative solvent-accessible surface area (SASA) variation values were calculated for lysozyme (blue) and CYP3A4 (green) conformations with respect to their equilibrated structure, considering 2 to 8 combined NM ensembles.

The comparison of standard MD and dpMDNM ensembles concerning their relative SS content and the relative SASA variation from the equilibrated structure is shown in Figure 10. For lysozyme (Figure 10, blue boxes), the relative SS content indicated a marginal loss of only 10%, with similar average values and slightly increased variations as more NMs were considered. We observed the same in the relative SASA variation except for the more pronounced increase in variations as more NMs were considered. On the other hand, the relative SS content of CYP3A4 (Figure 10, green boxes) was even smoother, with a loss of less than 5% and consistent average values across all ensembles. However, the relative SASA variation revealed a slight disparity between the standard MD and dpMDNM ensembles. Standard MD displayed increased values, averaging around 1.03, while dpMDNM presented lower average values of 0.99. This difference may be attributed to prolonged exposure to solvent and local motions in standard MD, allowing for greater solvent accommodation than dpMDNM. The RMSF of Cα atoms for lysozyme revealed that all dpMDNM ensembles exhibited higher values than standard MD, resembling the behavior observed in NMR ensembles (Figure S9, left panel). In the case of CYP3A4, the RMSF values were similar between standard MD and dpMDNM ensembles, except that the two peaks corresponding to loop regions of residues at positions around 200 and 275 exhibited much larger values in MD (Figure S9, right panel). In conclusion, when SS content, SASA variation, and RMSF analyses are considered, it can be inferred that the dpMDNM approach allows extensive conformational sampling for both proteins without compromising the structural quality of the conformations.

### 3.5 Energy Surface of the Normal Mode Space Using dpMDNM

**Figure 11:**
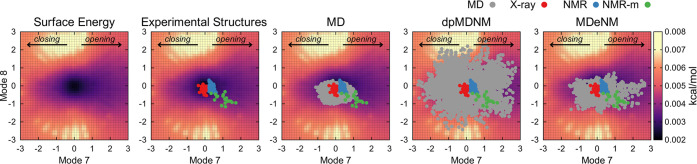
Restraining energy landscape in the surface spanned by modes 7 and 8 of lysozyme and the different plotted ensembles from experiments, standard MD, dpMDNM, and MDeNM. The ensemble of conformations in the latter two methods was obtained using 30 NM combined vectors based on modes 7 and 8. The restraining energetic cost to maintain the conformation at a particular position along a given vector was obtained using the VMOD module in CHARMM. The opening/closing motion directions are indicated for mode 7. The restraining energy values are shown by colors ranging from low (black) to high (yellow). X-ray (red), free-state NMR (blue), and bound-state NMR (green) conformations are indicated in the maps.

The dpVAC approach involves displacing the initial structure incrementally along an NM vector using harmonic restraining potentials (VMOD module in CHARMM) (Figures 3 and 4) in a vacuum. Originally, VMOD was designed to guide a system to a specified point along the vector under a harmonic restraining potential, forcing the structure to adopt a desired conformation.^73^ The displaced structure at a specific point along the NM combination vector is characterized by an energetic cost of maintaining that particular conformation, corresponding to the restraining energy. Therefore, it was possible to generate a bidimensional restraining energy surface map for lysozyme, illustrating the energetic cost of each conformation in the NM space using the set of 30 vectors combining modes 7 and 8 (Figure 11). This map reveals that the hinge-bending motion (mode 7) of lysozyme extends to larger distances with lower energy values in comparison to twisting (mode 8), with the opening movement (positive direction) being more energetically favorable. Notably, the movement along mode 8 becomes more extensive as the hinge-bending opening movement occurs. Interestingly, all experimental structures are in these low-energy regions, including the lysozyme bound-state (Figure 10, green points).

Conformations from standard MD, dpMDNM, and MDeNM ensembles were mapped onto the energy surface (Figure 11), showing that standard MD conformations are confined to low-energy regions around unbound structures, with structural displacements within −1.5 to 1.5 Å of mode 7 and −1.1 to 0.4 Å of mode 8. In contrast, dpMDNM could explore a larger conformational space, including higher energy regions (values around −2.5 to 2.5 Å of mode 7 and −2 to 2 Å of mode 8), covering all experimental structures. Similarly, MDeNM covered such structures, highlighting its capability to explore the conformational space within energetically accessible regions.^24^ A recent study comparing various hybrid methods found that in numerous cases, MDeNM outperformed several techniques, including coMD, ClustENM, and ClustENMD, for a more extensive sampling.^23^ In our comparison between dpMDNM and MDeNM, our findings suggest that dpMDNM is more efficient in exploring the NM space of lysozyme, extending the sampling to higher energy regions that could be of interest, particularly in the context of ligand binding (Figure 11). The extensive surface coverage observed in dpMDNM results from imposed positional restraints along the NM vectors, compelling the system to explore high-energy regions. As a result, dpMDNM has emerged as a promising alternative for conducting protein conformational sampling.

## 4 Conclusions

Developing efficient conformational sampling methods is of high interest in studying biomolecular systems, particularly in protein function.^16,23,24^ Computational techniques utilizing NMs are well-established in protein conformational sampling studies, combining NM vectors to explore the NM space.^24,54,55,75,120^ This work introduced a minimization algorithm to generate uniformly oriented NM vectors for wide protein conformational exploration. The exploration uses a novel distributed point Molecular Dynamics using Normal Modes (dpMDNM) approach, which integrates NM vector displacement and TMD complemented with standard MD simulations. This resulted in an extensive conformational exploration of lysozyme and CYP3A4, providing valuable insights into relevant structural aspects for a better understanding of the functions of these proteins.

We obtained a uniformly oriented set of vectors issued by the combination of several NMs until eight, a number deemed sufficient to cover both structural and functional aspects of proteins.^23^ The number of vectors can also be increased for a more extensive conformational exploration of the NM space, as observed when combining only two NMs. In addition, this work underscores the importance of using NMs with higher frequencies to cover a larger area in the lower part of the frequency range. Efficient conformational exploration of CYP3A4’s Channel 2 was obtained only when more NMs were combined. Although our work provides an alternative for assessing protein conformational properties considering a large number of NMs, linear NM combinations may fail to effectively capture complex conformational transitions in some cases. Strategies that involve recalculating NMs^122^ or updating the NM vector direction^123^ during simulations represent promising alternatives for smoothly accessing those states through NM vectors. These aspects can be incorporated to enhance the capabilities of the dpMDNM approach.

The methods developed in this work can be adapted to any NM vectors obtained from other techniques, such as ENM,^29−32^ rotation-translation-block (RTB),^124^ internal coordinate NMs (iNMA),^125^ nonlinear rigid block NM (NOLB),^126^ or even from principal component analysis obtained by standard MD simulations^56^ or experimental conformations.^75^ In addition, the ensemble of conformations generated by dpMDNM has the potential to improve the structural fitting of experimental conformation obtained from SAXS^127,128^ or cryo-EM^122,129^ techniques.

The dpMDNM approach offers a framework for protein conformational studies beyond simply exploring the NM space. By treating a set of combined NM vectors, one can compare them and identify neighbor vectors that describe potential pathways for targeted displacements, either toward a known conformation or uncharted regions of the NM space. Moreover, the capability of dpMDNM to quantify the energetic cost of displacing a protein along a specific NM vector becomes valuable. This feature can be useful when focusing on low-energy regions of the NM space, indicating likely pathways toward distinct conformations, as demonstrated in the case of lysozyme. Given the significant advancements in protein structure prediction achieved by AlphaFold^130^ and RosettaFold,^131^ high-confidence protein models are expected to accurately reproduce their intrinsic motions. The dpMDNM approach can potentially investigate the functional aspects of various other proteins or even those that have not yet been revealed through experimental methods.

## Data Availability

The input parameters and trajectories for lyzozyme and CYP3A4 are available at 10.5281/zenodo.13982000. The minimization algorithm, the selected sets of vectors used in this work, and a tutorial for performing dpMDNM using lysozyme are available at https://github.com/antonielgomes/dpMDNM.

## Supporting Information Available

Flowchart of the minimization algorithm; structural measurements of lysozyme and CYP3A4; RMSD values of experimental conformations of lysozyme and CYP3A4; RMSF of lysozyme experimental ensembles; conformational sampling of lysozyme in the structural space; structural aspects of CYP3A4’s Channel 2; conformational sampling of CYP3A4 in the structural space; membrane orientation of CYP3A4; RMSF calculations for lysozyme and CYP3A4; minimal distance of additional points; and relations between the hyperspherical space and normal mode spaces (PDF)
Minimization process in two dimensions (MP4)
Minimization process in three dimensions (MP4)
