# Supplementary material for: Extended Sampling of Macromolecular Conformations from Uniformly Distributed Points on Multidimensional Normal Mode Hyperspheres
Source: J Chem Theory Comput. 2024 Dec 12;20(24):10770–86. doi: 10.1021/acs.jctc.4c01054 (PMC11672670; doi:10.1021/acs.jctc.4c01054)
Supplement: Supplementary file 1 — ct4c01054_si_001.pdf [file ct4c01054_si_001.pdf]

Supporting information

Extended sampling of macromolecular conformations from uniformly distributed points on multidimensional normal mode hyperspheres

Antoniel A. S. Gomes[1,2,3], Mauricio G. S. Costa[4], Maxime Louet[3], Nicolas Floquet[3], Paulo M. Bisch[1], David Perahia[2]**

[1]Laboratório de Física Biológica, Instituto de Biofísica Carlos Chagas Filho, Universidade Federal do Rio de Janeiro, Rio de Janeiro 21941-902, Brasil

[2]Laboratoire de Biologie et Pharmacologie Appliquée (LBPA), UMR 8113, CNRS, École Normale Supérieure Paris-Saclay, Gif-sur-Yvette 91190, France

[3]Institut des Biomolecules Max Mousseron, UMR 5247, CNRS, Université de Montpellier, ENSCM, Montpellier, Cedex 05 34095, France

[4]Programa de Computação Científica, Vice-Presidência de Educação Informação e Comunicação, Fundação Oswaldo Cruz, Rio de Janeiro 21040-900, Brasil

*corresponding authors: antonielaugusto@gmail.com & david.perahia@ens-paris-saclay.fr

Supplementary Text

The minimization algorithm for dispersing points on the hypersphere's surface was implemented using Python's Sequential Least SQuares Programming (SLSQP) library. Initially, N points are randomly placed on the hypersphere's surface (see Fig. S1). Here is an overview of the steps involved in a minimization cycle: 1) Set the exponent s to 2 and the scaling factor α to the minimum pairwise distance between points. 2) Minimize the energy one position at a time by cycling through all the points in the system. This likely involves adjusting the positions of the points to find a configuration that minimizes the system's overall energy. 3) Update the parameters, increase the exponent s by a factor 2, and update the scaling factor α, using the minimum distance between the new positions; 4) Repeat the energy minimization (step 2) until the energy gradient (ΔE) reaches a low value. 5) Accept new positions only if the global energy is lower than the energy of the previous iteration, aiming to reach the global minimum. By iteratively performing these steps, the algorithm configures points on the hypersphere's surface with the lowest possible energy, ensuring a well-dispersed distribution.

An alternative approach addressed the increased complexity in higher-dimensional spaces, making it challenging to find the global minimum of the system. Thus, in systems with 4 or more dimensions, two steps in the algorithm were modified: step 2) Minimize only half of the points in the system; and step 3) Update the parameters and generate new positions by mirroring the minimized positions to obtain the other half of the points. The mirroring is performed by taking the diametrically opposing points on the hypersphere, allowing the algorithm to navigate higher-dimensional spaces, improving the likelihood of reaching the global minimum of the system.

Supplementary Figures

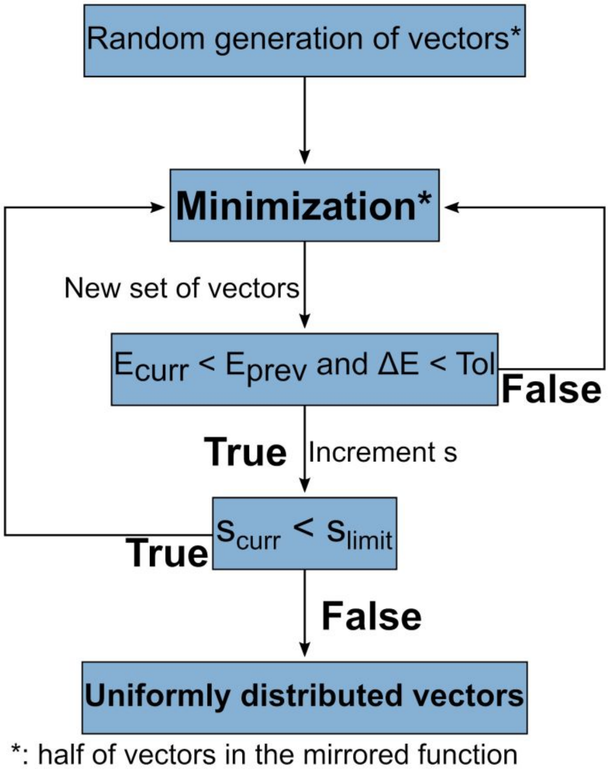

Figure S1. Flowchart of the minimization process to obtain uniformly distributed vectors.

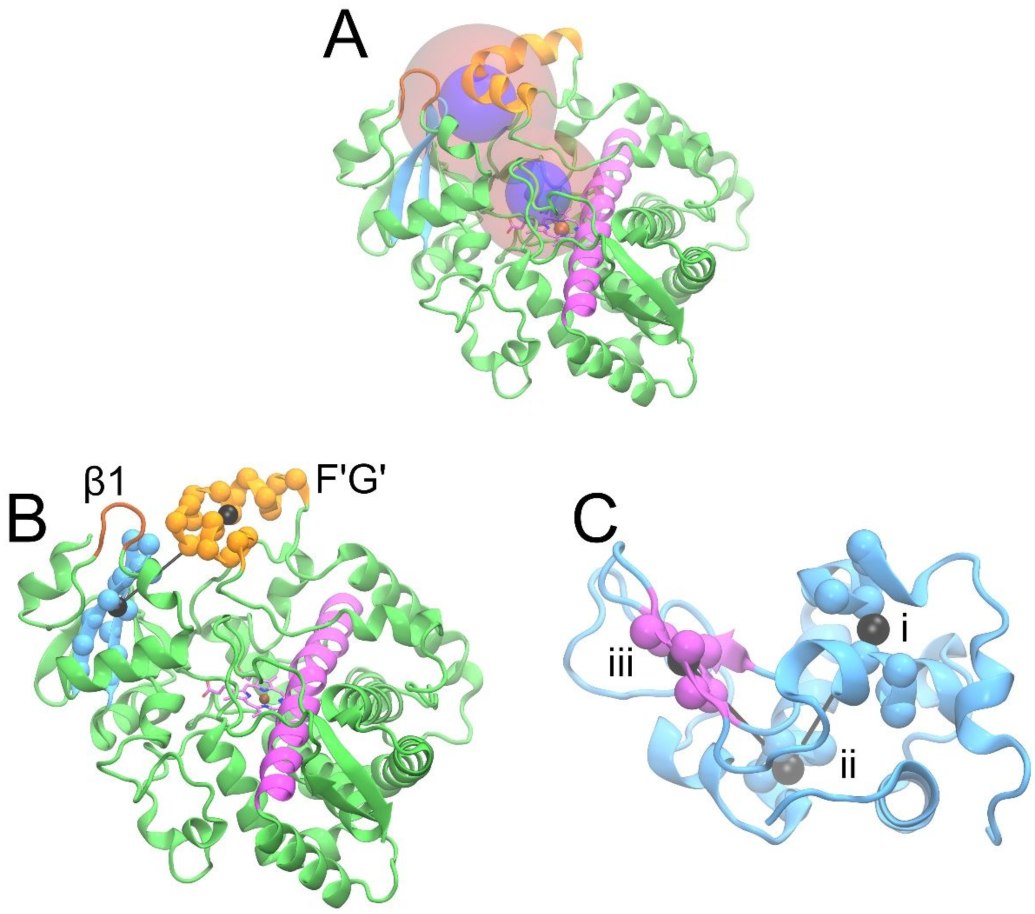

Figure S2. Structural measurements of Lyzozyme and CYP3A4. Lysozyme is depicted as a cyan cartoon, highlighting the β-sheet region as purple. CYP3A4 is depicted as a green cartoon, with highlighted regions including helices I (purple), F' and G' (orange), the sheet β1 (cyan), and the A-anchor (brown). Heme group and Fe^{2+} are represented as purple sticks and brown spheres, respectively. (A) Orientation of spheres capturing the volume dynamics of Channel 2, using Epock[1]. Blue and red spheres correspond to contiguous seeds and included regions, respectively. (B) The center of mass of Cα atoms from F'G' and β1 regions were considered to calculate the

F'G'-β1 distance. (C) The breathing angle of Lysozyme was calculated considering regions i, ii, and iii. The center of mass of each group of atoms is shown as black spheres.

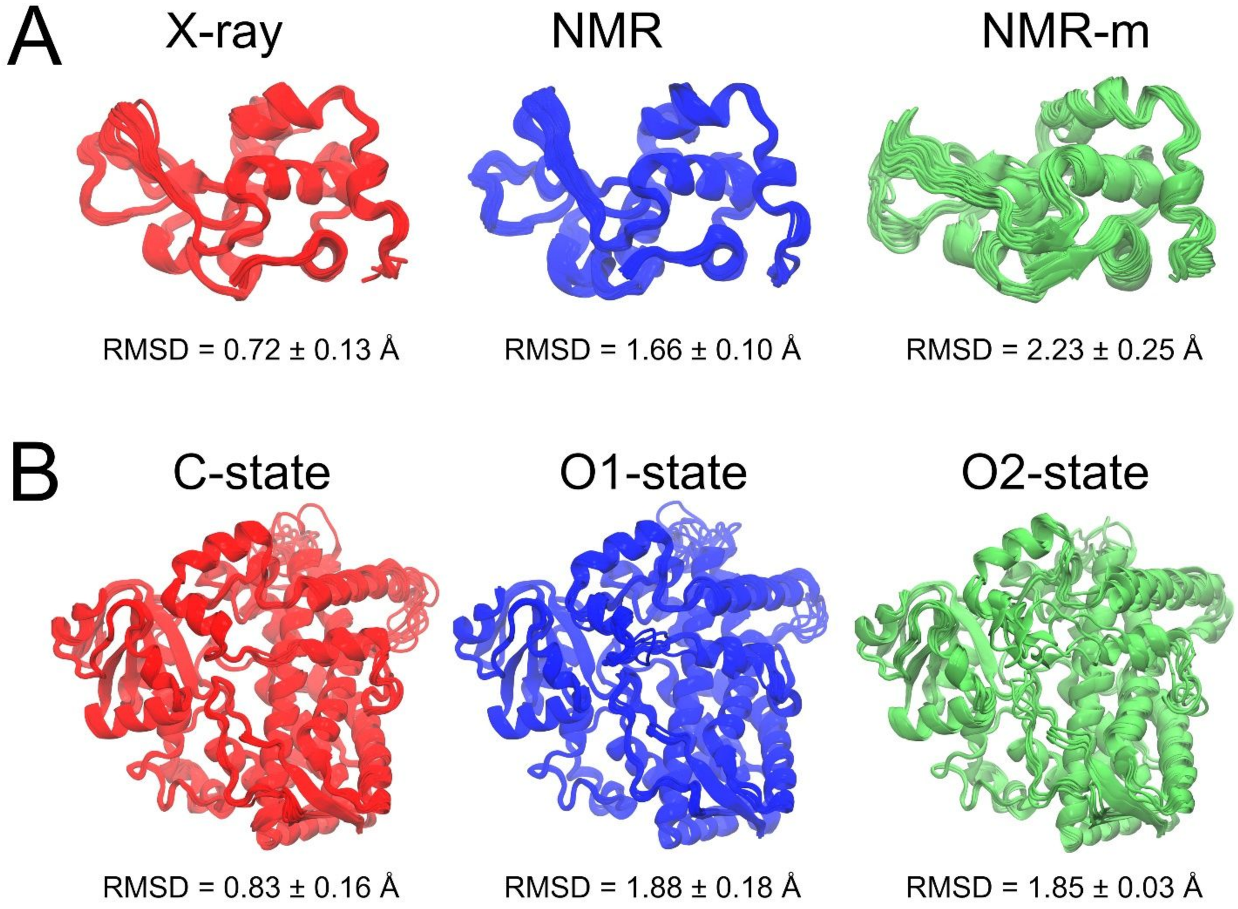

Figure S3. Root Mean Square Deviation (RMSD) of Lysozyme and CYP3A4 experimental ensembles. Average RMSD values and standard deviations were calculated using the minimized structures from our MD simulations data as a reference, considering the protein backbone atoms of each experimental ensemble. (A) Lysozyme ensembles corresponding to X-ray, free-state and bound-state NMR conformations are represented by red, blue, and green cartoons, respectively. (B) CYP3A4 ensembles corresponding to C-state, O1-state, and O2-state are represented by red, blue, and green cartoons, respectively.

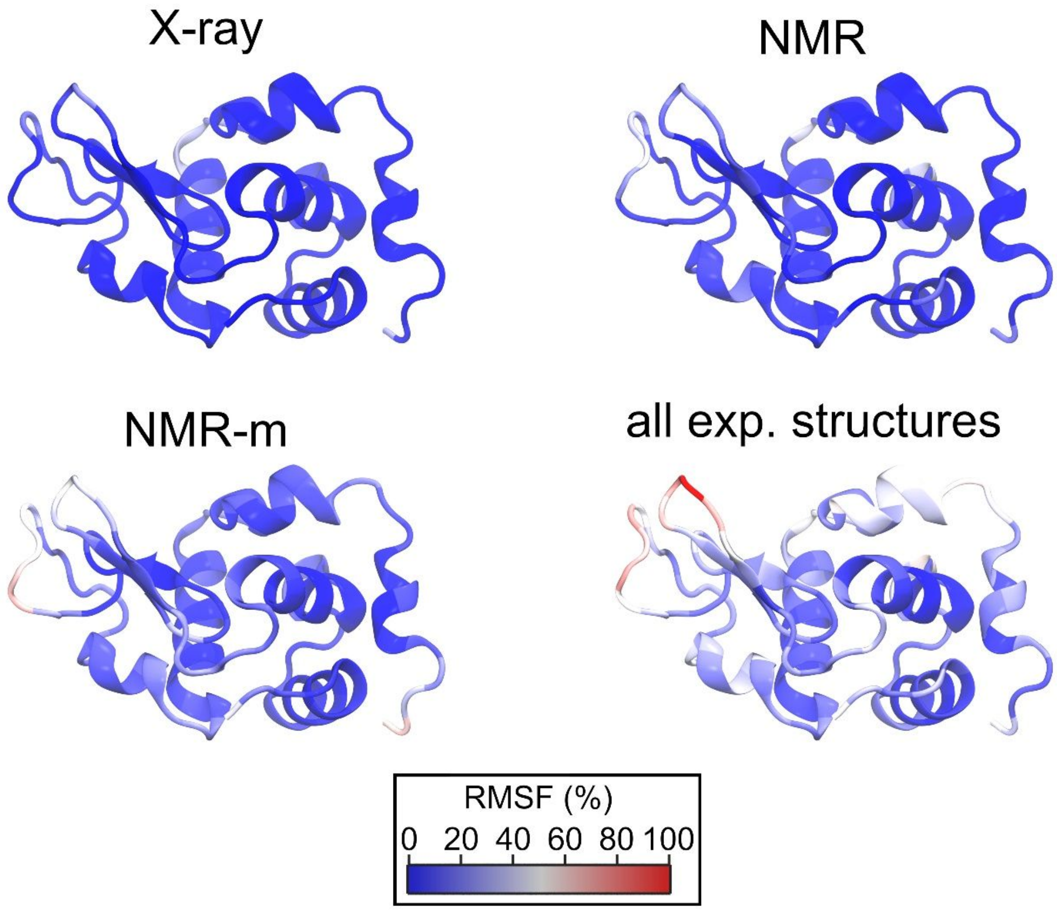

Figure S4. Root Mean Square Fluctuation (RMSF) of Lysozyme experimental ensembles. RMSF values were calculated for each residue and colored as a blue-to-red palette, representing low-to-high fluctuations in percentage.

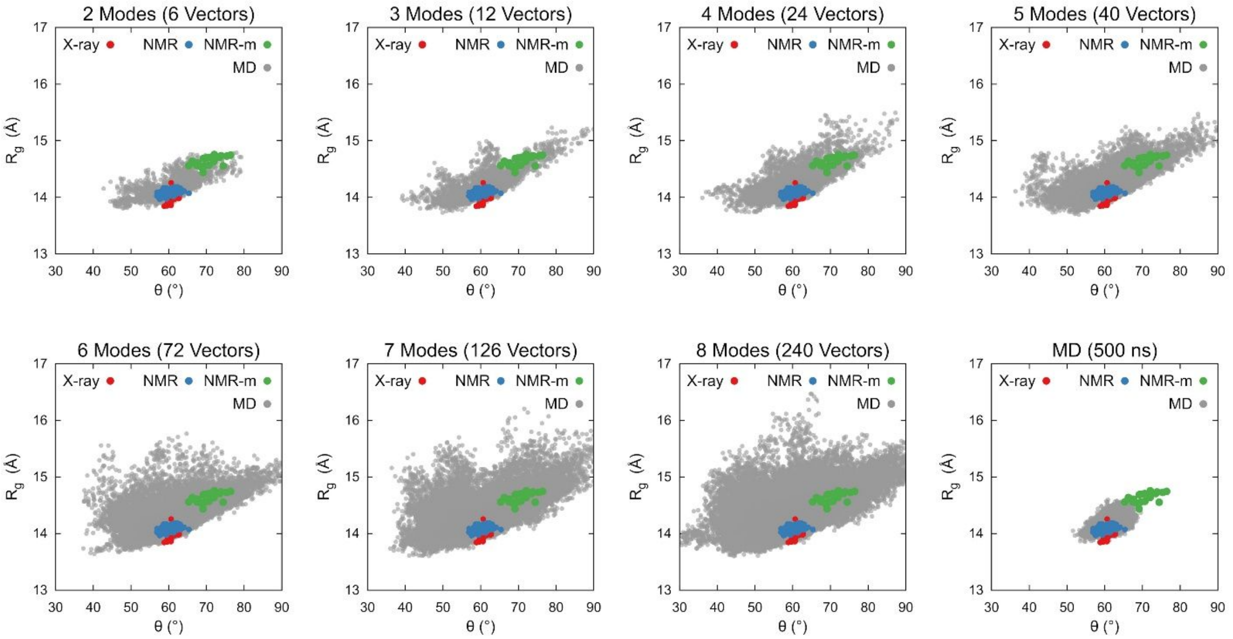

Figure S5. Conformational sampling of Lysozyme using dpMDNM within the structural space. The two-dimensional space is defined by the breathing angle (θ) and the radius of gyration (R_g). All the seven ensembles of conformations generated using different numbers of modes and vectors, along with those from standard MD, are depicted as gray circles. X-ray, free-state and bound-state NMR conformations are represented by red, blue, and green circles, respectively.

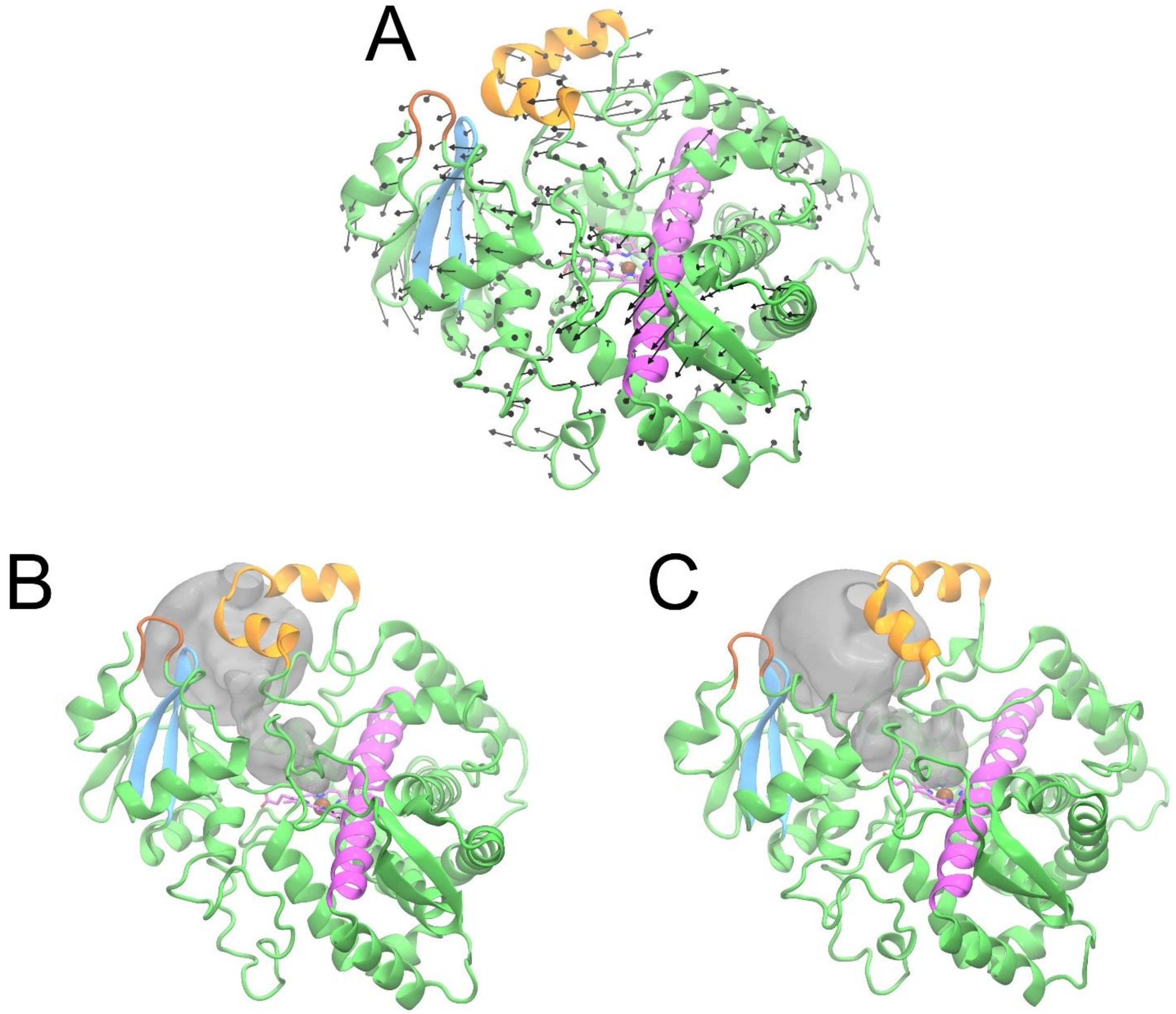

Figure S6. Structural aspects of CYP3A4's Channel 2. CYP3A4 is depicted as a green cartoon, with highlighted regions including helices I (purple), F' and G' (orange), the sheet β1 (cyan), and the A-anchor (brown). Heme group and Fe^{2+} are represented as purple sticks and brown spheres, respectively. (A) Atomic displacements of mode 11 are shown as black arrows, describing the opening of Channel 2. Representative conformations of CYP3A4 with the highest volume of Channel 2 obtained from (B) standard MD and (C) dpMDNM are shown.

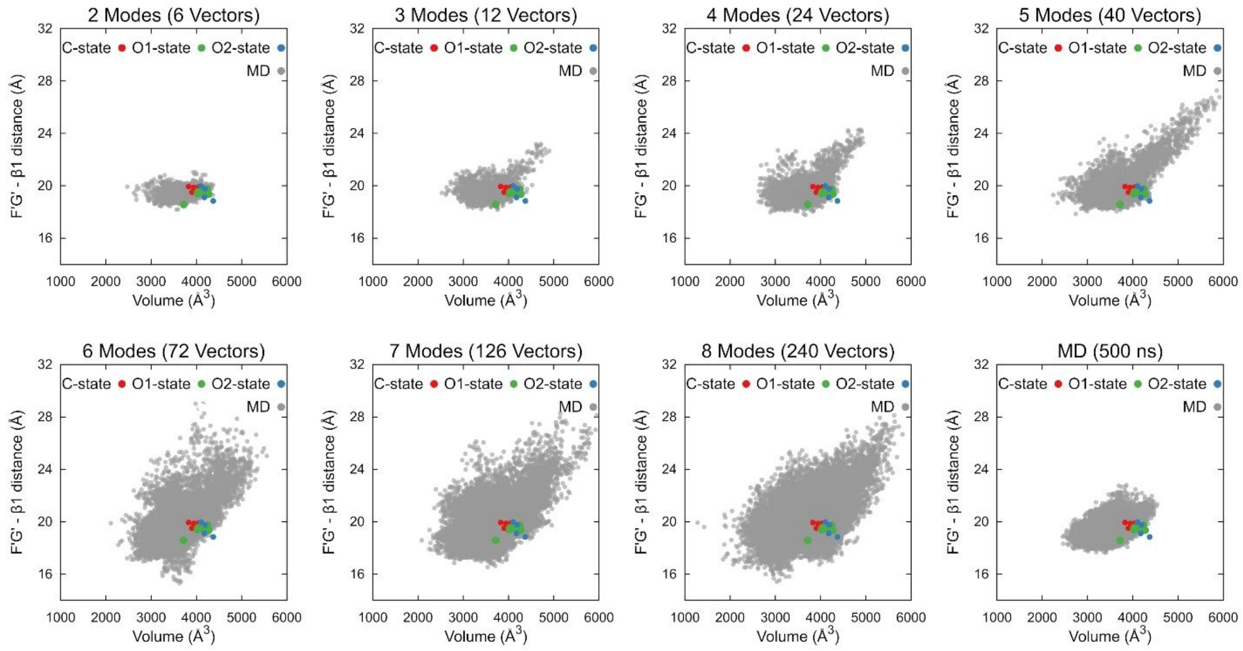

Figure S7. Conformational sampling of CYP3A4 using dpMDNM in the structural space. The bi-dimensional space is defined by the volume cavity of Channel 2 and the channel opening, measured as the distance between the F' and G' helices and β-1. All seven generated ensembles of conformations ranging from 2 to 8 combined NMs are mapped into the space of modes 7 and 8, and the ensemble of conformations from standard MD, are depicted as gray circles. The X-ray structures from C-, O1-, and O2-states are indicated by red, blue, and green circles, respectively.

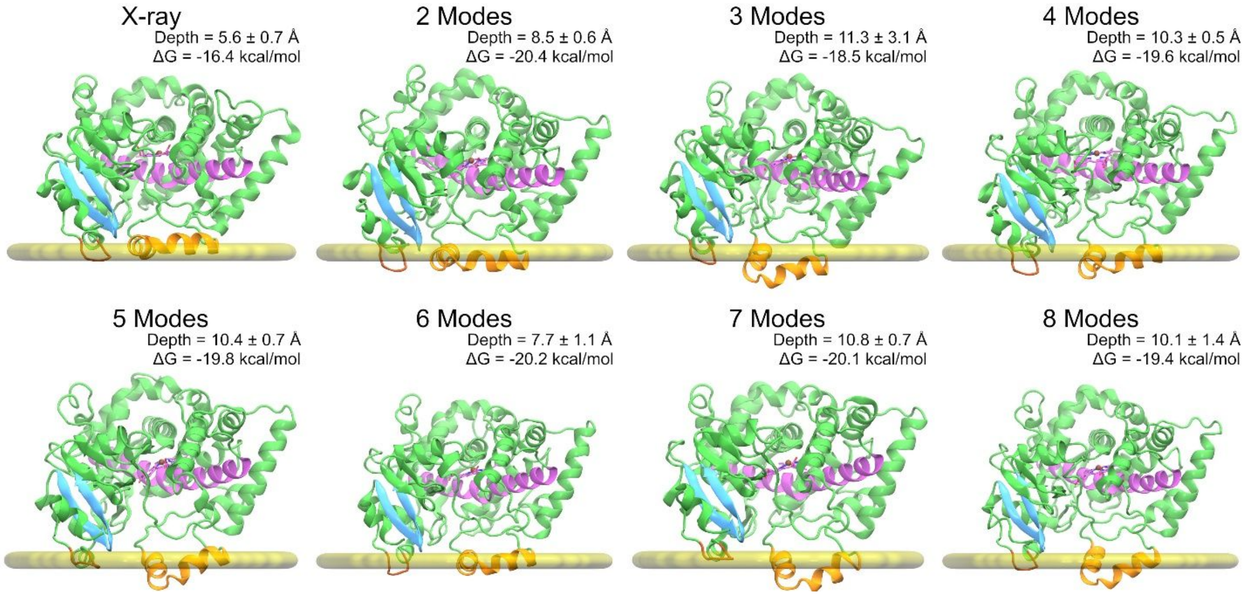

Figure S8. Prediction of the orientation of CYP3A4 on the lipid bilayer. Protein membrane penetration depth and predicted protein transfer energy from water to the lipid bilayer (ΔG) values were obtained from the Orientations of Proteins in Membranes (OPM) web server for the X-ray (initial structure) and representative structures from ensembles of 2 to 8 combined normal modes. The membrane is depicted as a yellow surface, while CYP3A4 is represented as a green cartoon, with the helices I (purple), F' and G' (orange), and the sheet β1 (cyan) and A-anchor (brown). The heme group and Fe^{2+} are shown as purple sticks and brown spheres, respectively.

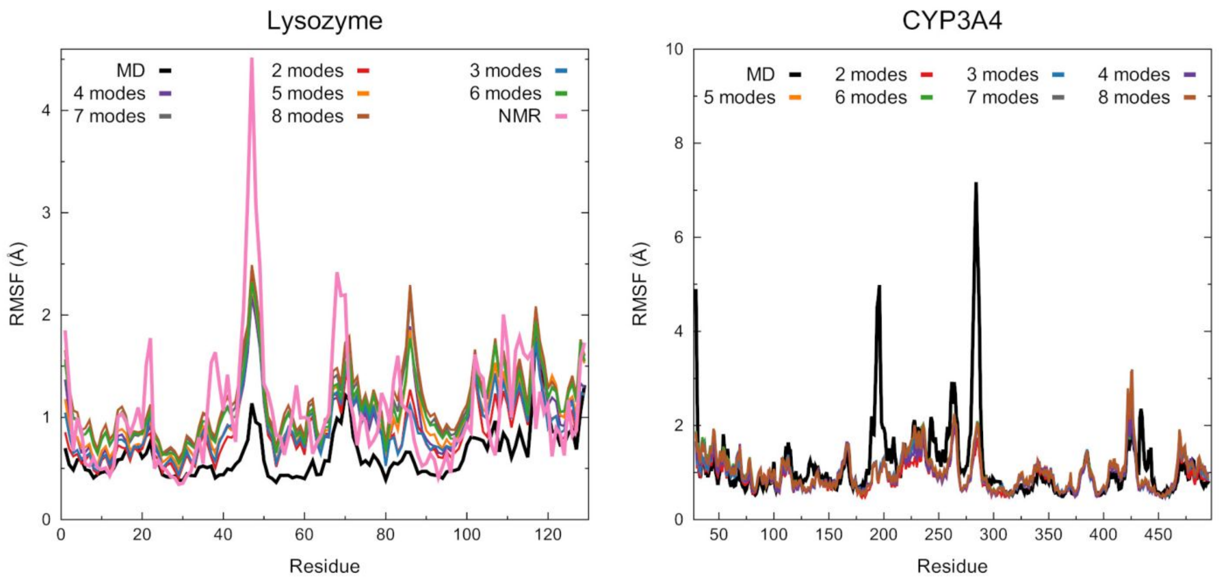

Figure S9. RMSF of Cα atoms for Lysozyme and CYP3A4. Calculations were performed for the seven ensembles of conformations (2 to 8 modes, from 7 to 14) and classical MD simulations. For Lysozyme, the NMR ensemble is also presented.

Supplementary Tables

Table S1. Minimal distance of points in 3 dimensions for additional cases. Values are compared to those found by Kottwitz[2].

Points	Mdist	Kottwitz
6	1.41421	–
7	1.25687	–
8	1.21556	–
9	1.15470	–
10	1.09143	–
11	1.05146	–
12	1.05146	–
13	0.95641	0.956414
14	0.93386	0.9338626
15	0.90266	0.902665
16	0.88057	0.880574
17	0.86244	0.862444
18	0.83821	0.838217
19	0.80856	0.808558
20	0.80438	0.804391

Table S2. Differences between the minimal distances in the hyperspherical and normal mode spaces, according to the relation between point distances and RMSD difference of displaced structures, and the Pearson correlation coefficient between the minimal distances in both spaces.

Dimensions/ Normal modes	Distance/RMSD difference		Correlation coefficient	
	Lysozyme	CYP3A4	Lysozyme	CYP3A4
2	0.00052 ± 0.00046	0.000076 ± 0.000031	0.999999423	0.999999998
3	0.00056 ± 0.00051	0.000085 ± 0.000046	0.999999016	0.999999992
4	0.00048 ± 0.00044	0.000106 ± 0.000071	0.999999116	0.999999977
5	0.00037 ± 0.00037	0.000094 ± 0.000069	0.999999246	0.999999974
6	0.00032 ± 0.00031	0.000084 ± 0.000064	0.999999398	0.999999975
7	0.00029 ± 0.00028	0.000078 ± 0.000059	0.999999463	0.999999976
8	0.00031 ± 0.00028	0.000070 ± 0.000053	0.999999418	0.999999978

Supplementary References

(1) Laurent, B.; Chavent, M.; Cragnolini, T.; Dahl, A. C. E.; Pasquali, S.; Derreumaux, P.; Sansom, M. S. P.; Baaden, M. Epock: Rapid Analysis of Protein Pocket Dynamics. *Bioinformatics* **2015**, *31* (9), 1478–1480. https://doi.org/10.1093/bioinformatics/btu822.

(2) Kottwitz, D. A. The Densest Packing of Equal Circles on a Sphere. *Acta Crystallogr A* **1991**, *47* (3), 158–165. https://doi.org/10.1107/S0108767390011370.
